# ﻿A deeper look into the diversity of *Phyllium* leaf insects from Indonesia: seven new species and two unique egg morphologies (Phasmatodea, Phylliidae)

**DOI:** 10.3897/zookeys.1256.162609

**Published:** 2025-10-23

**Authors:** Royce T. Cumming, Evelyn Marie Foley, Frank H. Hennemann, Stephane Le Tirant, Daawia, Evie Lilly Warikar, Heron Yando, Bambang Suhartawan, Katharina Henze, Thies H. Büscher, Sarah Bank

**Affiliations:** 1 Montreal Insectarium, Montréal, Québec, Canada Montreal Insectarium Montréal Canada; 2 Richard Gilder Graduate School, American Museum of Natural History, New York, NY, USA Richard Gilder Graduate School, American Museum of Natural History New York United States of America; 3 Biology, Graduate Center, City University of New York, New York, NY, USA City University of New York New York United States of America; 4 Department of Entomology, Rutgers, The State University of New Jersey, New Brunswick, NJ, USA The State University of New Jersey New Brunswick United States of America; 5 Department of Biology, Faculty of Mathematics and Natural Sciences, Cenderawasih University, Papua 99224, Indonesia Cenderawasih University Papua Indonesia; 6 Department of Environmental Engineering, Faculty of Civil Engineering and Planning, University of Science and Technology Jayapura, Jayapura, Indonesia University of Science and Technology Jayapura Jayapura Indonesia; 7 Department of Animal Evolution and Biodiversity, Johann-Friedrich-Blumenbach Institute of Zoology and Anthropology, University of Göttingen, Göttingen, Germany University of Göttingen Göttingen Germany; 8 Department of Functional Morphology and Biomechanics, Zoological Institute, Kiel University, Kiel, Germany Kiel University Kiel Germany

**Keywords:** Endemic, leaf insects, morphological counteradaptations, Phasmida, Phylliinae, Phylliini, Southeast Asia, walking leaf

## Abstract

Leaf insects of the genus *Phyllium* Illiger, 1798 are primarily distributed across Southeast Asia, with most species known from the Philippines, Malaysia, and Indonesia. Subsequent to a morphological and phylogenetic review of all available Indonesian *Phyllium*, seven previously unknown species were identified and are described herein as *Phyllium boislardi***sp. nov.**, from northern Kalimantan; *Phyllium cayabyabi***sp. nov.**, from northern Kalimantan; *Phyllium crapulatum***sp. nov.**, from western Kalimantan; *Phyllium hennemanni***sp. nov.**, from Sulawesi; *Phyllium illusorium***sp. nov.**, from Buton; *Phyllium morganae***sp. nov.**, from Yapen; and *Phyllium ouelleti***sp. nov.**, from Obi. In addition to describing seven new species, two previously undescribed egg morphotypes are identified within Phylliidae, bringing the total number of recognized phylliid egg morphotypes to 13. The twelfth morphotype, found in *Phyllium cayabyabi***sp. nov.**, is characterized by spatulate pinnae and hollow, columnar pinnae covering the entire egg surface. The thirteenth, observed in *Phyllium hennemanni***sp. nov.**, is unique among phylliids in possessing lateral flaps that are held slightly away from the egg capsule. Due to the pronounced sexual dimorphism in phylliids, and the importance of establishing new species within a phylogenetic context, as well as correctly matching male and female specimens, a comprehensive phylogenetic tree was constructed based on nearly all currently described *Phyllium* species. Furthermore, the conditions outlined in Article 23.9.1 of the International Code of Zoological Nomenclature 1999 (ICZN), specifically sub-articles 23.9.1.1 and 23.9.1.2, have been satisfied regarding the binomen *Phyllium longicorne* Latreille, 1802. This name is herein designated as **nomen oblitum**, in favor of the junior synonym *Pulchriphyllium bioculatum* (Gray, 1832), **nomen protectum**. Therefore, the name *Pulchriphyllium bioculatum* (Gray, 1832) is considered valid and protected in accordance with the International Code of Zoological Nomenclature article 23.9.2.

## ﻿Introduction

Phylliidae, commonly referred to as leaf insects or walking leaves, represent a lineage within Phasmatodea well known for their exceptional leaf mimicry. This morphological adaptation serves as a highly effective camouflage mechanism, allowing these insects to remain concealed within the forest canopy. Currently, the family includes 114 extant species (Brock et al. 2025) and one extinct species (Wedmann et al. 2007), though continued exploration, particularly in under-sampled regions such as Indonesia, regularly yields new taxa (Cumming et al. 2020a; Seow-Choen 2021, 2023). Phylliids exhibit marked sexual dimorphism: females are larger and flightless whereas males are smaller and capable of flight and occasionally attracted to artificial lights at night (Bank and Bradler 2022; Boisseau et al. 2022). With females being flightless and given their arboreal habitat, it makes it difficult for researchers to reach and study them. However, occasionally with severe weather events or within anthropogenically disturbed habitats where lower canopy foliage facilitates human detection, females become more apparent (Brock and Hasenpusch 2002; Cumming et al. 2021).

Taxonomic classification and phylogenetic understanding of the Phylliidae have been historically limited due to morphological convergence and inadequate sampling across their range (Bank et al. 2021b). Morphological stasis in adult forms—both among allopatric and sympatric populations—has further complicated species delimitation (Cumming et al. 2021). In contrast, phylliid egg morphology has significant diversity, even between closely related taxa, and offers valuable diagnostic traits (Clark 1978, 1979; Clark-Sellick 1997; Cumming et al. 2019a; Bank et al. 2021b; Büscher et al. 2023). Eggs exhibit diverse morphotypes, defined by structures such as exochorionic ribs, pinnae, and overall shape, with eleven general types recently recognized within the Phylliidae (Büscher et al. 2023). Additionally, egg surfaces may bear moisture-activated adhesive compounds adapted to varying substrate types (Büscher et al. 2023).

Geographically, the Phylliidae are restricted primarily to Southeast Asia (Seychelles in the west, Tibet to the north, Fiji to the east, and New Caledonia to the south). The complex geological history of Southeast Asia and the Indo-Australian Archipelago has created a mosaic of biogeographical regions that have profoundly influenced patterns of species diversification and distribution (Lohman et al. 2011; Toussaint et al. 2020). Wallacea, in particular, represents a transitional zone bounded by Wallace’s and Lydekker’s lines of faunal balance and is characterized by high levels of endemism and biotic turnover (Rowe et al. 2019; Letsch et al. 2020). With phylliids being highly camouflaged, sexually dimorphic, and typically canopy-dwelling, biogeographic structuring can remain cryptic without integrative taxonomic approaches. Molecular phylogenetics, combined with detailed morphological analysis, offers a powerful means of disentangling these evolutionary relationships and tracing dispersal and speciation events across this fragmented landscape (Condamine et al. 2015; Bank et al. 2021a, 2021b).

In this study, we integrate morphological analysis and molecular phylogenetics to describe seven new species of *Phyllium* from across the Indonesian archipelago elucidating multiple independent colonization events, putative dispersal/restrictive corridors, and the evolutionary consequences of geographic isolation within Indonesia. Within these seven herein described new species, we also illustrate two new egg morphotypes, never before seen in the phylliids. To contextualize these new taxa within the broader evolutionary framework of Phylliidae, we constructed a near-comprehensive phylogeny using molecular sequence data. This approach not only improves resolution of interspecific relationships but also ensures the correct pairing of sexually dimorphic males and females.

## ﻿Materials and methods

### ﻿Specimens

Specimens for this study come from museum collections and records from several private collections. All specimens were legally exported from Indonesia to their various current locations following Indonesian wildlife export laws. Additionally, besides reviewing specimens in person, we also utilized the high-quality images of captive reared specimens as well as many type material images available publicly online (www.phasmatodea.com, www.phasmida.speciesfile.org) in order to better understand intraspecific variability presented within species. Measurements of specimens were made to the nearest 0.1 mm using digital calipers and are given for individual specimens. Holotype and paratype specimens herein designated are deposited within several different institutional and private collections which are explicitly listed within the type material information of the new species descriptions where the following collection acronyms are used:

**ICZN** International Code of Zoological Nomenclature [ISBN 0 85301 006 4]

**IMQC** Insectarium de Montréal, Montréal, Québec, Canada

**MNHN**Muséum National d’Histoire Naturelle, Paris, France

**ZSM**Zoologische Staatssammlung München [Munich], Germany

**Coll FH** Private collection of Frank H. Hennemann, Germany

**Coll RC** Private collection of Royce T. Cumming, California, USA

**Coll SS** Private collection of Sigetake Suzuki, Japan

**Coll TB** Private collection of Thies H. Büscher, Germany

Morphological abbreviations utilized within include (listed morphologically anterior to posterior)

**Sc** subcosta

**R** radius

**R1** radius 1

**R2** radius 2

**R3** radius 3

**Rs** radial sector

**R–M** radius to media crossvein

**M** media

**MA** media anterior

**MP1** first media posterior

**MP2** second media posterior

**M–Cu** media to cubitus crossvein

**Cu** cubitus

**CuA** cubitus anterior

**CuP** cubitus posterior

**1A** first anal

### ﻿Photography

Photographs of specimens deposited within the IMQC collection were taken by René Limoges using a Nikon D850 DSLR camera (Nikon Corporation, Tokyo, Japan) with Nikon Micro-Nikkor 200mm f/4 lens on Manfrotto 454 micrometric positioning sliding plate (Manfrotto, Casolla, Italy). Lighting was provided by two Nikon SB-25 flash units with a Cameron Digital diffusion photo box (Henry’s, Vancouver, Canada). Adobe Photoshop Elements 13 (Adobe Inc., San Jose, USA) was used as post-processing software. Additional photographs which are not from the IMQC are explicitly listed within the figure captions with citation to the photographers.

Photos of specimens from the ZSM and Coll FH collections were taken with a Nikon D7000 camera equipped with a Nikon DX AF-SMacro 40 mm lens and a wireless Nikon SU-800 dual speed light system. Photos were stacked with a Helicon® FB tube and the corresponding Helicon® focus stacking software (Helicon Focus Bracketing Tube Configuration Utility, v. 25.06.08; produced by HeliconSoft; Kharkiv, Ukraine).

### ﻿Scanning electron microscopy (SEM)

Eggs and antennae were investigated using SEM. Micrographs were taken from dried samples, sputter coated with 10 nm gold/palladium (Leica Bal-TEC SCD500, Leica Camera AG, Wetzlar, Germany). For overviews, a Hitachi TM3000 (Hitachi High-technologies Corp., Tokyo, Japan) at 15 kV acceleration voltage was used, employing a rotatable specimen holder on which the samples were mounted on a short insect pin (Pohl 2010). Detailed micrographs of antennae were obtained using the Hitachi S4800 at an acceleration voltage of 5 kV. Adobe Photoshop Elements 13 (Adobe Inc., San Jose, USA) was used as post processing software for cropping and contrast adjustment.

### ﻿Phylogenetic analysis

Using the taxon and gene sampling of Bank et al. (2021b), we selected all 42 taxa of *Phyllium* along with four *Cryptophyllium* species to serve as outgroup. Four of the therein undescribed *Phyllium* species have since been described (*Phyllium bankae*, *Phyllium iyadaon*, *Phyllium ortizi* and *Phyllium samarense*; Cumming et al. 2023, 2025). Following their protocol (Bank et al. 2021b), we complemented the dataset with Sanger-sequence data for a maximum of six loci (16S, 18S, 28S, COI, COII, H3) for an additional 12 specimens from Indonesia (Buton, Kalimantan, Peleng, Sulawesi and Yapen) and deposited the data in GenBank (Suppl. material 1).

Sequences for each locus were aligned using mafft v. 7.526 (--maxiterate 100000 --globalpair) (Katoh and Standley 2013) followed by gap removal and concatenation as described elsewhere (Bank et al. 2021a). The resulting supermatrix was partitioned by gene into six data blocks. We performed a partitioned phylogenetic analysis with a random starting tree in IQ-TREE v. 2.3.2 using the extended model selection and allowing partitions to be merged (-m MFP and --merge greedy) (Nguyen et al. 2015; Chernomor et al. 2016; Kalyaanamoorthy et al. 2017; Minh et al. 2020). Node support was assessed using Ultrafast Bootstrap (UFBoot) and the single branch test (SH-aLRT) with 10,000 replicates each (Guindon et al. 2010; Hoang et al. 2018).

## ﻿Results

Among Phylliidae, *Phyllium* Illiger, 1798 is currently the most species-rich genus with 35 species recognized (Brock et al. 2025). Most of these species have been described in the last decade, and new ones continue to be discovered frequently (e.g., *Phyllium bankae*; Cumming et al. 2025). One species, “*Phyllium longicorne* Latreille, 1802” is herein designated as nomen oblitum (discussed further below) and seven newly described species of *Phyllium* are named and illustrated below, bringing the total number of species in the genus to 41.

### ﻿Checklist to *Phyllium* Illiger, 1798 species and their general geographic distributions

1. *Phyllium antonkozlovi* Cumming, 2017 (in Cumming et al. 2017a) [Philippines: Luzon]
2. *Phyllium arthurchungi* Seow-Choen, 2016 [Malaysia: Sabah]
3. *Phyllium bankae*Cumming et al., 2025 [Indonesia: Halmahera]
4. *Phyllium bilobatum* Gray, 1843 [Philippines: (vague locality only)]
5. *Phyllium boislardi* sp. nov. [Indonesia: northern Kalimantan]
6. *Phyllium bonifacioi* Lit & Eusebio, 2014 [Philippines: Luzon]
7. *Phyllium bourquei* Cumming & Le Tirant, 2017 (in Cumming et al. 2017a) [Philippines: Luzon]
8. *Phyllium bradleri* Seow-Choen, 2017 [Malaysia: Sabah]
9. *Phyllium brossardi*Cumming et al., 2017b [Malaysia: Sabah]
10. *Phyllium cayabyabi* sp. nov. [Indonesia: northern Kalimantan]
11. *Phyllium chenqiae* Seow-Choen, 2017 [Malaysia: Sabah]
12. *Phyllium conlei* Cumming, Valero & Teemsma, 2018b [Indonesia: Lombok]
13. *Phyllium crapulatum* sp. nov. [Indonesia: western Kalimantan]
14. *Phyllium cummingi* Seow-Choen, 2017 [Malaysia: Sabah]
15. *Phyllium elegans* Größer, 1991 [Papua New Guinea: New Britain]
16. *Phyllium ericoriai*Hennemann et al., 2009 [Philippines: Batan, Luzon, Marinduque, Catanduanes, Sibuyan]
17. *Phyllium fallorum* Cumming, 2017 [Philippines: Mindanao]
18. *Phyllium gantungense*Hennemann et al., 2009 [Philippines: Palawan]
19. *Phyllium gardabagusi* Cumming, Bank, Le Tirant & Bradler, 2020a [Indonesia: Java]
20. *Phyllium hausleithneri* Brock, 1999 [Malaysia: West Malaysia (Peninsular)]
21. *Phyllium hennemanni* sp. nov. [Indonesia: Sulawesi]
22. *Phyllium illusorium* sp. nov. [Indonesia: Buton]
23. *Phyllium iyadaon*Cumming et al., 2023 [Philippines: Mindoro]
24. *Phyllium jacobsoni* Rehn & Rehn, 1934 [Indonesia: Java]
25. *Phyllium letiranti* Cumming & Teemsma, 2018 [Indonesia: Peleng, Taliabu, Sanana (= Sulabesi; earlier name Xulla Besi; Goodall, 1943)]
26. *Phyllium mabantai* Bresseel et al., 2009 [Philippines: Mindanao]
27. *Phyllium mamasaense* Größer, 2008 [Indonesia: Sulawesi]
28. *Phyllium mindorense*Hennemann et al., 2009 [Philippines: Mindoro]
29. *Phyllium morganae* sp. nov. [Indonesia: Yapen]
30. *Phyllium nisus* Cumming, Bank, Le Tirant & Bradler, 2020a [Indonesia: Sumatra]
31. *Phyllium ortizi*Cumming et al., 2023 [Philippines: Mindanao]
32. *Phyllium ouelleti* sp. nov. [Indonesia: Obi]
33. *Phyllium palawanense* Größer, 2001 [Philippines: Palawan]
34. *Phyllium philippinicum*Hennemann et al., 2009 [Philippines: Luzon]
35. *Phyllium rubrum* Cumming, Le Tirant & Teemsma, 2018b [Malaysia: West Malaysia (Peninsular)]
36. *Phyllium saltonae* Cumming, Baker, Le Tirant & Marshall, 2020b [Philippines: Palawan]
37. *Phyllium samarense*Cumming et al., 2023 [Philippines: Samar]
38. *Phyllium siccifolium* (Linnæus, 1758) [Indonesia: Buru, Ambon, Seram]
39. *Phyllium telnovi* Brock, 2014 [Indonesia: West Papua Province]
40. *Phyllium tobeloense tobeloense* Größer, 2007 [Indonesia: Halmahera]

1. *Phyllium tobeloense bhaskarai* Cumming, Le Tirant & Hennemann, 2019a [Indonesia: Morotai]

1. *Phyllium woodi* Rehn & Rehn, 1934 [Philippines: Sibuyan]

### ﻿*Phyllium longicorne* Latreille, 1802, nomen oblitum

Historic phylliid publications include an early work of Latreille (1802), with a short note about “siccifolia Lin.” where Latreille describes the female *Phyllium siccifolium*, and a very general male leaf insect description (“Antennes longues, sétacées; articles nombreux et alongés. Ailes dépassant les élytres” which roughly translates to “antennae long, setaceous; numerous and elongated articles. Wings extending beyond the elytra”) and naming it “*Phyllium longicorne*. Latr.” (Latreille 1802). This binomial has escaped almost all authors since this publication and has largely been ignored. This is likely due to the publication of Guérin-Méneville (1838) which stated that the sexual dimorphism misled Latreille and caused him to name the male as a separate species. This vague synonymization by Guérin-Méneville (1838) is likely what led to the name being ignored for subsequent decades. Inadvertently, the name *Phyllium longicorne* was so forgotten that it evaded all authors (including lists of synonyms) and was not rediscovered and published until Brock and Büscher (2022), where it was included in a checklist of species. Following the rediscovery of this name, and thanks to the efforts of Emmanuel Delfosse (France), a historic male specimen was located in the MNHN collection, which is on a specimen pin of the correct style for the era (early 1800’s), and the specimen is labeled in Latreille’s own handwriting with a male sex symbol on one label and “Latreille” on the second. As this specimen is in the correct collection, matches the vague morphological description given by Latreille (1802), and is labeled in his own hand, it is assumed to be the *Phyllium longicorne* specimen in question [specimen observable here: https://phasmida.speciesfile.org/otus/852807/overview]. Upon reviewing the specimen, we have identified it as a male *Pulchriphyllium bioculatum*, which poses a problem as the name *Pulchriphyllium bioculatum* is younger (1832), and therefore according to the ICZN would be a junior synonym of *Phyllium longicorne* which would have priority due to its age (1802). To ensure stability of the nomenclature, we apply the ICZN ruling for such forgotten names (ICZN article 23.9.1.).

This presents the first case of a nomen oblitum within Phylliidae as the corresponding ICZN conditions have been met: 1) This species of Latreille’s is a senior synonym of *Pulchriphyllium bioculatum* (the Latreille specimen does not appear to be an undescribed taxon, in which case nomen oblitum would not apply). 2) The name *Phyllium longicorne* has not been used as a valid name after 1899 (ICZN art. 23.9.1.1.) as the only sources which have been found that state this name are the original Latreille (1802) publication, a verbatim reprint of his work in 1820 by Buffon (Latreille 1820), and the vague synonymization by Guérin-Méneville (1838). 3) Preferred junior synonym (*Pulchriphyllium bioculatum*) is in wide use and has been so for the last 193 years where it has been used by 50+ different authors and has appeared in more than 100 different publications (Brock et al. 2025).

Following this reasoning, the International Code of Zoological Nomenclature (ICZN) article 23.9.1. conditions (23.9.1.1. and 23.9.1.2.) have been met regarding the binomial *Phyllium longicorne* Latreille, 1802, nomen oblitum in favor of the younger name *Pulchriphyllium bioculatum* (Gray, 1832), nomen protectum. Therefore, the name *Pulchriphyllium bioculatum* (Gray, 1832) is considered valid and protected in accordance with the International Code of Zoological Nomenclature article 23.9.2.

### ﻿Phylogenetic analysis

Obtaining and combining the molecular data resulted in a concatenated supermatrix comprising 58 taxa and 4573 nucleotide positions (Suppl. material 2). ModelFinder, implemented in IQ-TREE (Kalyaanamoorthy et al. 2017; Minh et al. 2020), merged the six locus-based data blocks into four partitions and selected the following best-fit models: 16S (GTR+F+I+G4), 18S (GTR+F+I+G4), 28S + H3 (TVM+F+I+G4) and COI + COII (GTR+F+I+R4).

**Figure 1.**
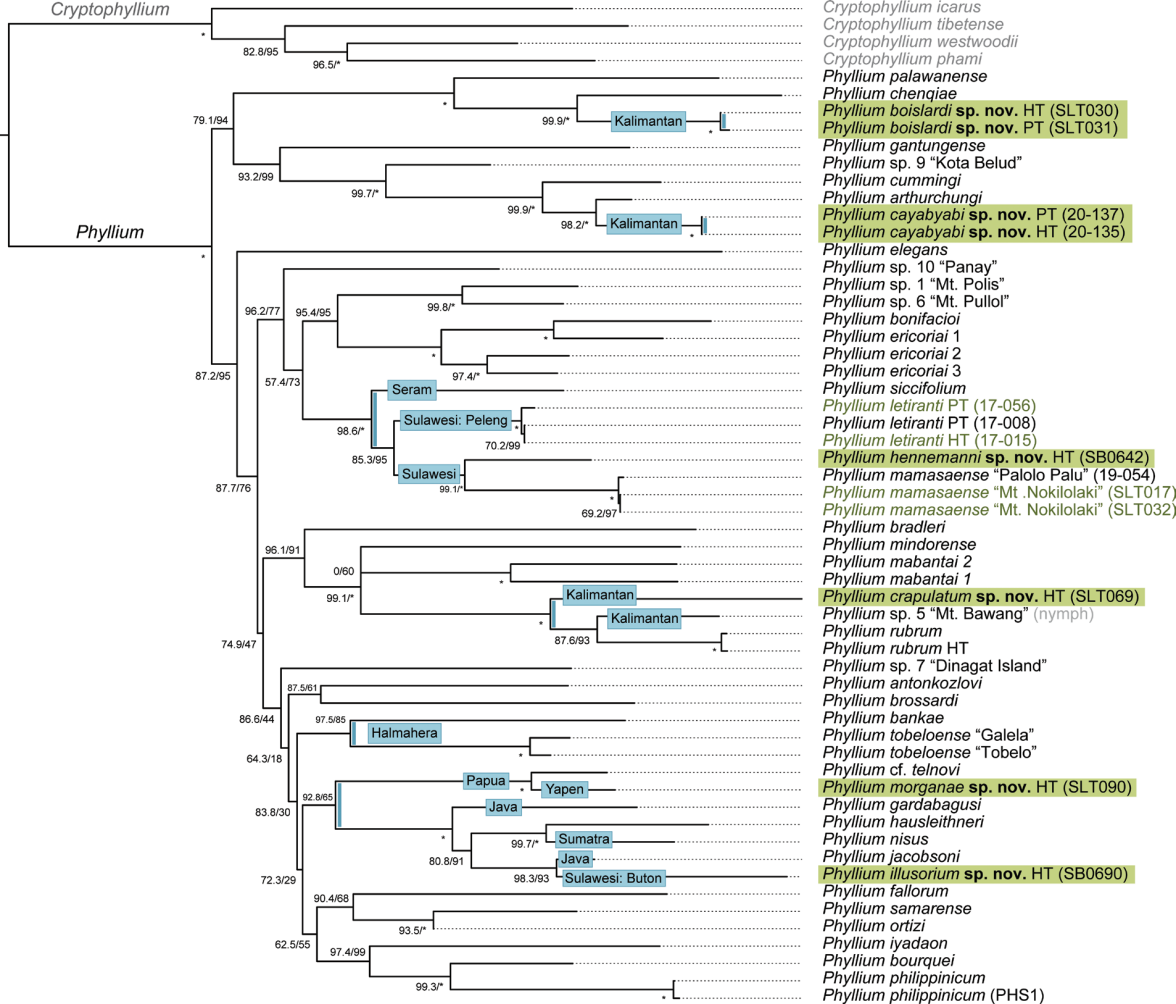
Maximum Likelihood tree with focus on the phylogenetic relationships of 54 *Phyllium* specimens. Tree is rooted with *Cryptophyllium* as the outgroup. Support values are shown at each node (SH-aLRT/UFBoot). The asterisk (*) indicates maximum support (100). Newly added specimens are shown in green text and new species are highlighted in green. The blue stripes on internal branches mark dispersal to Indonesia, with blue boxes specifying the exact island or region. Abbreviations: HT, holotype; PT, paratype.

The resulting Maximum Likelihood tree (Fig. 1, Suppl. material 3) is not entirely congruent with the phylogeny obtained in previous analyses (Bank et al. 2021b; Cumming et al. 2023), although this may be explained by the difference in outgroup sampling and phylogenetic inference. This is also reflected by the overall moderate support values, especially regarding the deeper nodes, while more shallow nodes are generally well supported.

**Figure 2.**
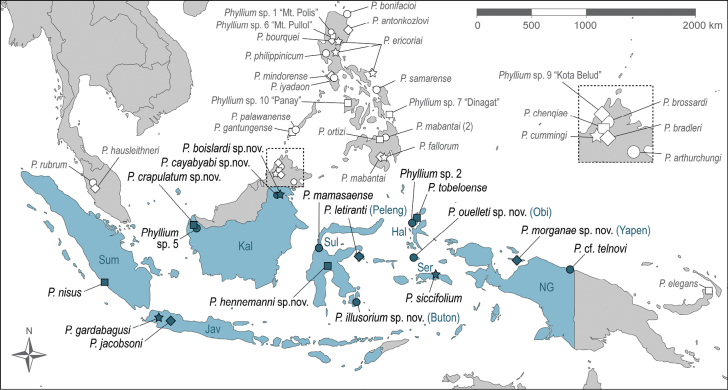
Distribution map for all *Phyllium* species used in the molecular analysis as well as the newly described species from Obi Island not represented in the phylogenetic tree. Species names of *Phyllium* from Malaysia, the Philippine Islands, and New Britain (Papua New Guinea) are shown in gray. The Indonesian archipelagic state is highlighted in blue and symbols of sampling sites of Indonesian *Phyllium* species are in dark blue. White symbols show the location of the remaining *Phyllium* from Malaysia, the Philippine Islands and New Britain (Papua New Guinea). The inset presents an enlarged section of Sabah (Malaysia: Borneo) and the six species found there. Abbreviations: Hal, Halmahera; Jav, Java; Kal, Kalimantan; NG, New Guinea (Papua); Ser, Seram; Sul, Sulawesi; Sum, Sumatra.

The additional specimens of *Phyllium letiranti* and *Phyllium mamasaense* are found to cluster together with their conspecifics. The other newly added specimens were molecularly confirmed to represent new species. *Phyllium hennemanni* sp. nov. from South Sulawesi was inferred as sister taxon to the three specimens of *Phyllium mamasaense* from Central Sulawesi. Both species form the sister group to *Phyllium letiranti* from the neighboring island of Peleng. The specimen from South of Sulawesi, *Phyllium illusorium* sp. nov. from Buton Island, appears to be unrelated to this clade, however. It is recovered as sister group to *Phyllium jacobsoni* from Java, with other close relatives from Java and Sumatra.

The four specimens from Malinau (North Kalimantan) were recovered as two unrelated species, *Phyllium boislardi* sp. nov. and *Phyllium cayabyabi* sp. nov. Both species, respectively, are sister group to species from Sabah (Malaysia). The other species from Kalimantan, *Phyllium crapulatum* sp. nov. from Mount Bawang (West Kalimantan), was not found to be conspecific with the unidentified nymph (*Phyllium* sp. 5) from the same location included in Bank et al. (2021b), but instead represents the sister group to this specimen and *Phyllium rubrum* (Peninsular Malaysia).

*Phyllium morganae* sp. nov. from Yapen Island is recovered as sister group to Phyllium cf. telnovi from Papua, a clade that is closely related to the lineage from Java/Sumatra. Whether the holotype of *Phyllium telnovi* from West Papua is conspecific with the specimen used in this analysis or is in fact closer related to *Phyllium morganae* sp. nov. will need to be addressed in future studies.

### ﻿Morphological results

Among the herein described new species, two exhibit previously undocumented egg morphotypes, bringing the total number recognized in Phylliidae to 13. The eggs of *Phyllium cayabyabi* sp. nov. are characterized by hollow, columnar pinnae covering the surface, while those of *Phyllium hennemanni* sp. nov. bear large, laterally projecting flaps—a structure not wholly unknown in the Phasmatodea (e.g., some species of *Trachythorax* Redtenbacher, 1908 have lateral flaps; however, these are not particularly comparable to *Phyllium hennemanni* sp. nov. eggs, as discussed further below in the morphological discussion section) and potentially linked to anti-parasitic functions (Bresseel and Constant 2021)).

In addition to these two new species with novel egg morphotypes, five other undescribed species have been identified as unique from congenerics. In several cases these species were morphologically difficult to differentiate based on our current, incomplete morphological knowledge as only a single sex is presently known (e.g., *Phyllium illusorium* sp. nov. is only known from male specimens which morphologically look very similar to *Phyllium jacobsoni*, in this case the geographic distance and the significant molecular divergence (Fig. 1) of these two species warranted their differentiation). Herein, only *Phyllium cayabyabi* sp. nov. was described from both male and female specimens; for all other herein described species morphological knowledge is fragmentary. Thankfully, most species were able to be included in the molecular analysis, thus allowing future discoveries such as opposite sexes to be properly linked to the species in question and move ever closer to the goal of more complete morphological knowledge (adult male, adult female, freshly hatched nymphs, and egg morphology).

### ﻿Taxonomy

**Phylliidae, Phylliinae, Phylliini**

#### ﻿ Phyllium

Taxon classificationAnimaliaPhasmatodeaPhylliidae

Illiger, 1798

0EA356D5-712E-5886-86BB-4E39CB33E1EE

##### Type species.

*Phyllium siccifolium* (Linnæus, 1758); type locality: ‘Indies’.

#### ﻿ Phyllium boislardi

Taxon classificationAnimaliaPhasmatodeaPhylliidae

Cumming, Foley, Hennemann, Le Tirant & Büscher sp. nov.

81311371-E6DF-568D-A8BA-8EE75FEC0263

https://zoobank.org/85608DD1-5EC8-4BD4-AA76-96AA53BE7D3B

Figs 3, 4, 5

##### Type material.

***Holotype*** (♀): Indonesia • N. Kalimantan, Malinau, V.2021, Local Coll. via Edy Bhaskara; DNA Sample: SLT030; Coll RC #21-037 [IMQC]. ***Paratypes***: (1♀, 1 egg): (1♀) Indonesia • N. Kalimantan, Malinau, III.2019, Local Coll. via Edy Bhaskara; DNA Sample: SLT030; Coll RC #21-036 [Coll RC]; (1 egg) Laid by the holotype female from Indonesia • N. Kalimantan, Malinau, V.2021, local coll. via Edy Bhaskara, Coll RC #22-001 [Coll RC].

##### Differentiation.

**Figure 3.**
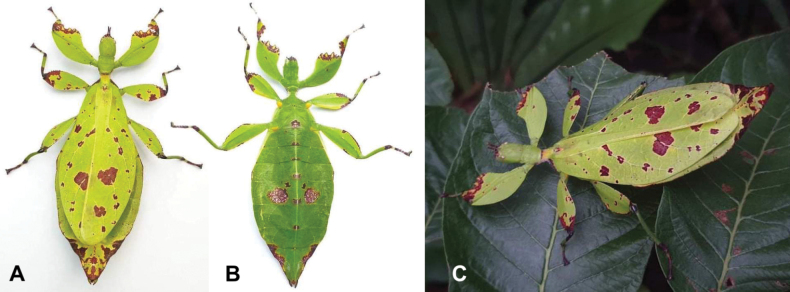
Live *Phyllium boislardi* sp. nov. holotype female [Coll RC #21-037] (images courtesy of Edy Bhaskara (Indonesia). A. Dorsal habitus; B. Ventral habitus; C. In situ, dorsolateral view.

**Figure 4.**
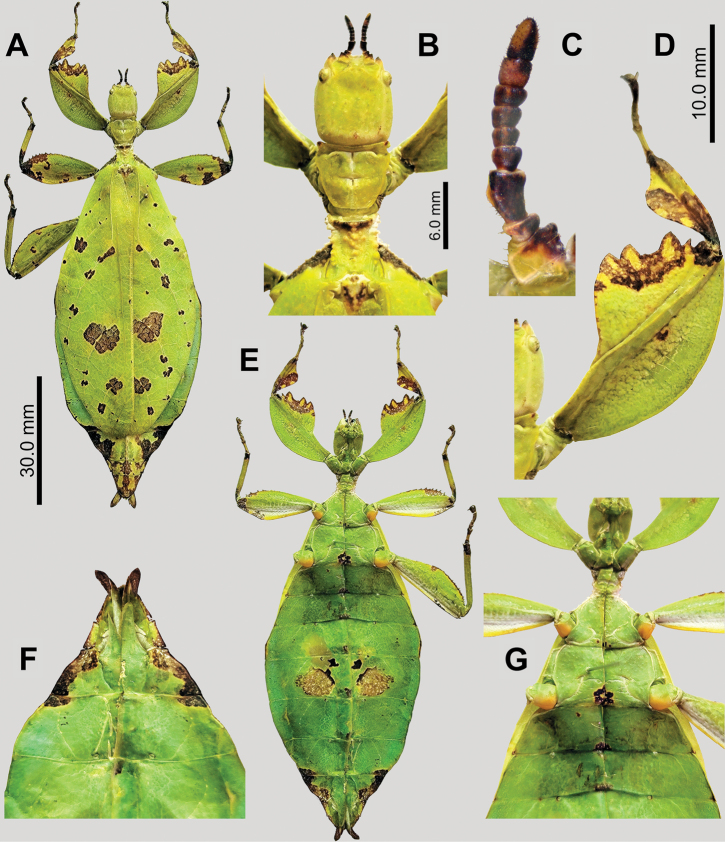
*Phyllium boislardi* sp. nov. holotype female Coll RC #21-037 [IMQC]. A. Dorsal habitus; B. Detail of antennae, head, and thorax, dorsal; C. Right antenna, dorsal; D. Detail of front right leg, dorsal; E. Ventral habitus; F. Terminalia, ventral (note that the subgenital plate was cut during the gutting process for preservation so it is distorted in the image); G. Thorax, coxae, and first abdominal sternites, ventral.

Male unknown. Female *Phyllium boislardi* sp. nov. (Fig. 3) are most similar to *Phyllium palawanense* due to similar femoral lobe shapes/serration and mesoprescutum nodes arranged in a somewhat haphazard way, not exclusively aligned along the sagittal plane. *Phyllium boislardi* sp. nov. can be differentiated from *Phyllium palawanense* by the mesopleurae, as *Phyllium palawanense* has mesopleurae which are marked with more prominent tubercles, vs *Phyllium boislardi* sp. nov. which has mesopleurae marked with smaller granulation (Fig. 4B). An additional feature which might also be useful is the ventral coxae coloration as *Phyllium boislardi* sp. nov. has pale orange coloration (Fig. 3B) vs *Phyllium palawanense* which has a pale pink color.

**Figure 5.**
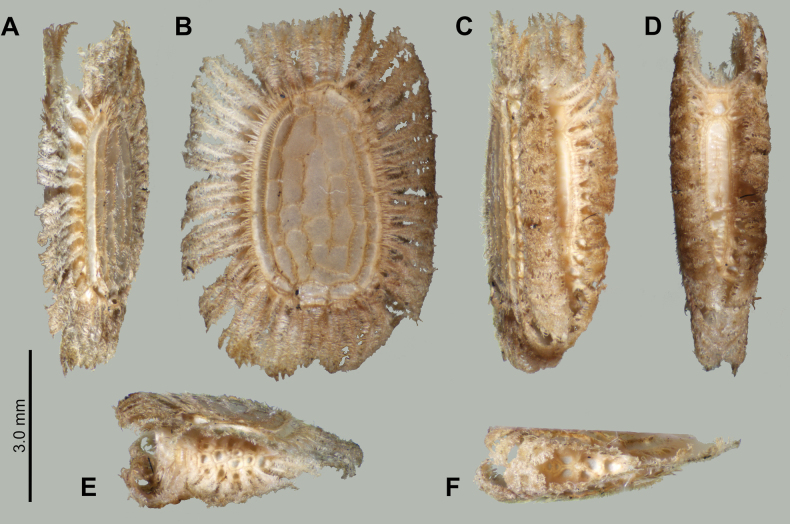
*Phyllium boislardi* sp. nov. paratype egg, Coll RC #22-001 [Coll RC]. Scale bar: 3.0 mm. A. Ventral view; B. Lateral surface; C. Dorsolateral view; D. Dorsal surface; E. Opercular (anterior) view; F. Posterior view.

Eggs of *Phyllium boislardi* sp. nov. (Fig. 5) are very similar to *Phyllium palawanense* eggs with the triangular cross-section and the large feather-like pinnae around the margins and operculum. These species can be differentiated by the differing micropylar plate shape as *Phyllium boislardi* sp. nov. has a thin, straight sided plate (Fig. 5D), vs *Phyllium palawanense* which has a wider, more ovoid plate. Additionally, the bald patches on the lateral walls of the capsule differ slightly, with *Phyllium palawanense* having longitudinal bald stripes vs *Phyllium boislardi* sp. nov. which has these longitudinal stripes broken up into subcells (Fig. 5B).

##### Description.

**Female. *Coloration*.** Coloration description is based upon images of the living type specimens (Fig. 3). In the holotype and paratype, the general coloration is pale green throughout. In the holotype, these are numerous colored patches across the dorsal and ventral surfaces (Fig. 3A) while the paratype is simply pale green throughout and lacks these colored patches. In the holotype, there are brown spots of varying sizes and shapes on the legs, abdomen, and tegmina. Ventral coxae coloration is pale orange (Fig. 3B).

***Morphology*. *Head*** capsule slightly longer than wide, with a dorsal surface that is smooth, lacking granulation (Fig. 4B). The posteromedial tubercle is present, singularly lobed, but not very prominent (Fig. 4B). Frontal convexity broad and ending in a blunted point; there are only a few short setae across the surface. Compound eyes small and only slightly protruding from the head capsule, not bulbous, taking up ~1/5 of the head capsule lateral margins (Fig. 4B). Ocelli absent. Antennal field approx. as wide as first antennomere.

***Antennae*** consist of nine segments (Fig. 4C), with the terminal segment not particularly wide or long (slightly shorter than the combined length of the previous two segments combined lengths). Antennomeres IX and the distal 1/2 of segment VIII have a rough, fuzzy texture with short, dark setae. The remaining segments are smooth, and sparsely marked with short, transparent setae, none prominent (Fig. 4C).

***Thorax*.** Pronotum with slightly concave anterior margin and lateral margins that anteriorly start wide, angle inward strongly towards the posterior margin which is slightly wider than 1/2 of the anterior margin width (Fig. 4B). The pronotum anterior margin has a prominent rim, the lateral margins are less prominent, and the posterior margin is weakly developed. The pronotum surface is relatively smooth, has a small but prominent sagittal slit in the center and another near the anterior. The remainder of the surface is smooth and marked with a few other furrows (Fig. 4B). Prosternum and the anterior 1/2 of the mesosternum are covered throughout by moderately spaced granulation. The posterior 1/2 of the mesosternum and the metasternum are relatively smooth (Fig. 4G). Mesoprescutum slightly longer than wide, lateral rims lumpy with 8–10 variable sized and spaced nodes (3–5 of these are somewhat prominent; Fig. 4B). Mesoprescutum anterior rim distinctly raised and slightly lumpy but not forming a distinct sagittal spine (Fig. 4B). Mesoprescutum surface wrinkled and lumpy, mostly just with nodes along the sagittal plane, but there are several prominent nodes spread throughout the surface (Fig. 4B). Mesopleurae begin to diverge ~½ of the way along the mesoprescutum, angle prominently away with straight margins (Fig. 4B). Mesopleurae lateral margins with five or six small spiniform tubercles with interspersed granulation throughout, giving the margin a roughly textured appearance since none are particularly prominent (Fig. 4B). Face of the mesopleura slightly wrinkled along the lateral margins, and marked with two notable divots, one on the anterior margin and one near the middle (Fig. 4B).

***Wings*.** Tegmina long, reaching the anterior margin of abdominal segment VIII. Tegmina venation; the subcosta (Sc) is the first vein in the forewing, running parallel with the margin for the first 1/2, and then bending and running towards the margin. The subcosta runs for ~¼ of the tegmina length. The radius (R) spans the anterior ½ of the forewing with two subparallel branched veins; the first radius (R1) branches ~¼ of the way through the wing length and terminates ~2/5 of the way through the wing length; the radial sector (Rs) branches ~1/3 of the way through the wing length and terminates near the distal 3/5 of the wing length. There is a weak continuation of the radius following the prominent Rs branching which continues on as a short but distinct R–M crossvein that weakly connects the two veins. The media (M) is bifurcate with both the media anterior (MA) and media posterior (MP) terminating near to the posterior of the tegmina. The cubitus (Cu) is also bifurcate, branching near the posterior ¼ of the wing into the cubitus anterior (CuA) and cubitus posterior (CuP) which both terminate near the wing apex. The first anal vein (1A) is simple and fuses with the cubitus ~1/5 of the way through the tegmina length. Alae vestigial nubs.

***Abdomen*.** Abdominal segments II through the anterior ½ of IV gradually and uniformly diverging. The posterior ½ of segment IV through the anterior ½ of segment VII are gradually converging. The posterior ½ of segment VII through the apex of the abdomen is converging to a blunted apex.

***Genitalia*.** Subgenital plate starts at the anterior margin of tergum VIII, is relatively narrow, and extends half-way onto tergum X. The overall shape is rather narrow, with the apex a blunted point (Fig. 4F). Gonapophyses VIII are long and narrow, slightly exceeding the apex of abdominal tergum X; gonapophyses IX are mostly obstructed from view (Fig. 4F). Cerci flat, and somewhat paddle-shaped, with narrow bases broadening out to a wide distal ½ (Fig. 4F).

***Legs*.** Profemoral exterior lobe broad, rounded, arching from end to end, with a width similar to the width of the interior lobe (Fig. 4D). Margin of profemoral exterior lobe smooth (Fig. 4D). Profemoral interior lobe ~2.5 × as wide as the greatest width of the profemoral shaft, slightly obtusely angled, and marked with several teeth (starting on the proximal end: two large triangular teeth, then a large gap, another large triangular tooth, followed by a smaller finely pointed tooth on the distal end; Fig. 4D). Mesofemoral exterior lobe approx. a narrow, rounded triangle with the greatest width similar to the mesofemoral shaft width, and the greatest width situated slightly distal to the midlength. Mesofemoral exterior lobe margin smooth, lacking teeth. Mesofemoral interior lobe is approx. the same width as the mesofemoral shaft, with a shape that is gently arching from end to end, with the distal 1/2 marked with 6–9 small, serrate teeth. Metafemoral interior lobe narrow on the proximal 1/2 with the wider distal 1/2 arching to the distal end. The distal 1/2 of the metafemoral interior lobe is marked with eight or nine small, serrate teeth. Metafemoral exterior lobe is thin and smooth, hugging the metafemoral shaft and lacks dentation. Protibia lacking an exterior lobe (Fig. 4D). Protibia interior lobe spans the entire length of the protibia and is ~2 × the width of the protibia shaft itself. The lobe is roundly triangular with the widest portion slightly situated slightly distal to the midlength. Mesotibiae and metatibiae simple, lacking exterior and interior lobes.

***Measurements*** (mm). Holotype, female: body length (including cerci and head, excluding antennae): 97.9, length/width of head: 8.4/7.2, antennae: 4.8, pronotum: 5.9, mesonotum: 8.6, length of tegmina: 62.8, greatest width of abdomen: 35.8, profemora: 20.2, mesofemora: 17.9, metafemora: 22.2, protibia: 11.4, mesotibia: 11.9, metatibia: 17.1.

***Measurements*** (mm). Paratype, female: body length (including cerci and head, excluding antennae): 91.1, length/width of head: 8.1/7.0, antennae: 4.5, pronotum: 6.0, mesonotum: 8.1, length of tegmina: 58.3, greatest width of abdomen: 35.3, profemora: 16.9, mesofemora: 15.8, metafemora: 19.5, protibia: 9.3, mesotibia: 10.4, metatibia: 16.2.

**Eggs** (Fig. 5). Margins rimmed with prominent feather-like pinnae. When viewed laterally, the general shape is ovoid (Fig. 5B), and in cross-section the egg is a narrow triangle (Fig. 5E, F). Lateral surfaces flattened and marked with seven longitudinal lines of bald impressions, the lines between appearing like a network of hairy ridges. Micropylar plate slender with relatively straight margins, and covering most of the length of dorsal egg surface. Micropylar cup small and situated on the posterior 1/3. Operculum somewhat teardrop shaped due to the triangular egg cross-section, flat, and with the lateral and ventral margins set with a row of the same long feather-like appendages seen along the longitudinal margins of the capsule (dorsal margin of the operculum lacks these pinnae). The operculum surface is marked throughout with irregularly sized pits with smooth, thick rims abutting the thick rim of the neighboring pits rims (Fig. 5E). The posterior of the egg capsule has similar pits to those found on the operculum but they are slightly larger and fewer (Fig. 5F). General color tan.

##### Measurements including the extended pinnae [mm].

 Length (including operculum expansion): 7.1; maximum width of capsule when viewed from lateral aspect 5.1; length of micropylar plate 3.4.

##### Etymology.

Patronym; named to honor Thierry Boislard (Canada), a colleague of the Montreal Insectarium and a good friend to the fourth author.

##### Distribution.

At present only known from the type locality of Malinau, in North Kalimantan, Indonesia (Fig. 2).

#### ﻿ Phyllium cayabyabi

Taxon classificationAnimaliaPhasmatodeaPhylliidae

Cumming, Foley, Hennemann, Le Tirant & Büscher sp. nov.

CE7CFC04-5E7A-562B-9792-0ED659BA3E63

https://zoobank.org/832724CC-E45D-4D03-B075-2B83C5F26390

Figs 6, 7, 8, 9, 10

##### Type material.

***Holotype*** (♀): Indonesia • North Kalimantan, Malinau Regency, Tanjung Lapang Village, August 2020, Coll RC #20-135 [IMQC]. ***Paratypes***: (2♀♀, 1♂, 1♀ nymph, 3 eggs): (1♀ nymph, 1♂) Indonesia • North Kalimantan, Malinau Regency, Tanjung Lapang Village, August 2020 (1♂) Coll RC #20-137; ♀ nymph #20-136 [Coll RC]. (2 eggs) Indonesia • North Kalimantan, Malinau Regency, Tanjung Lapang Village, August 2020; #20-138, #20-139 [Coll RC]; (1 egg): Indonesia • North Kalimantan, Malinau Regency, Tanjung Lapang Village, August 2020 [Coll TB]; (2♀♀): Indonesia • North Kalimantan, Malinau Regency, Tanjung Lapang Village, August 2020 [IMQC].

##### Differentiation.

**Figure 6.**
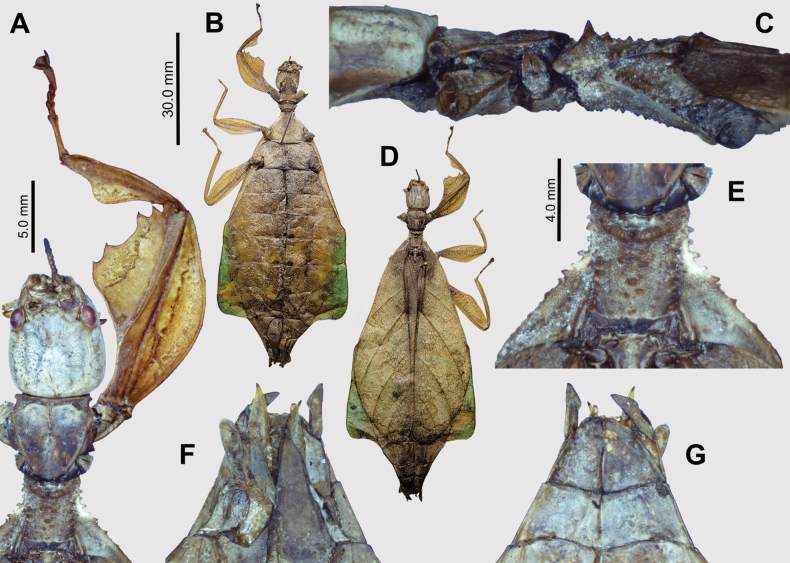
*Phyllium cayabyabi* sp. nov. holotype female, #20-135 (IMQC). A. Details of antenna, head, thorax, and front leg, dorsal; B. Habitus, ventral; C. Thorax, lateral (head to the left); D. Habitus, dorsal; E. Details of mesonotum, dorsal; F. Terminalia, ventral (note that the subgenital plate has been cut in half when it was gutted and dried); G. Terminalia, dorsal. Scale bar: 30.0 mm (B, D).

Female *Phyllium cayabyabi* sp. nov. (Fig. 6) are most morphologically similar to *Phyllium gantungense* and *Phyllium arthurchungi* (likely also similar to the *Phyllium cummingi* female, but that sex is not yet known at this time so no direct comparison can be made) due to similar femoral lobe shapes/serration and boxy abdominal morphology. *Phyllium cayabyabi* sp. nov. can be differentiated from *Phyllium gantungense* by the ventral coxae coloration, as *Phyllium gantungense* has distinctly black ventral coxae coloration while *Phyllium cayabyabi* sp. nov. does not have distinct black spots (unfortunately the exact color of the ventral meta- and mesocoxae cannot be determined as the type specimens were poorly preserved and the coloration did not survive well enough to discern). *Phyllium cayabyabi* sp. nov. can be differentiated from *Phyllium arthurchungi* by the arrangement of nodes along the mesoprescutum as *Phyllium arthurchungi* has the nodes exclusively situated along the sagittal plane in a tight line, while *Phyllium cayabyabi* sp. nov. has the nodes only generally along the sagittal plane, meandering side to side of the central line (Fig. 6E).

**Figure 7.**
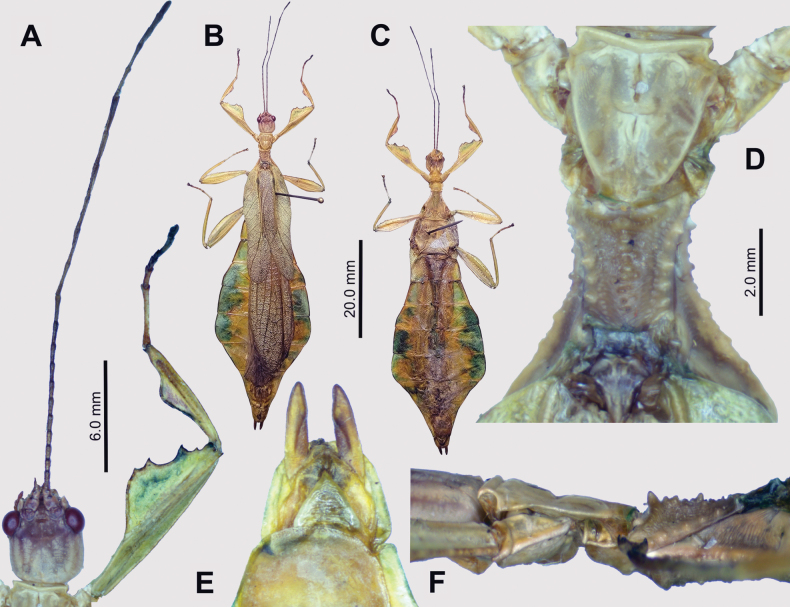
*Phyllium cayabyabi* sp. nov. paratype male, #20-137 (Coll RC). A. Details of antenna and head, dorsal; B. Habitus, dorsal; C. Habitus, ventral; D. Details of pronotum and mesonotum, dorsal; E. Terminalia, ventral; F. Thorax, lateral (head to the left). Scale bar: 20.0 mm (B, C).

Male *Phyllium cayabyabi* sp. nov. (Fig. 7) are similar to *Phyllium arthurchungi*, *Phyllium cummingi*, and *Phyllium gantungense* due to similar tegmina venation/length, the thorax shape and spination, the lobes of the legs, and the broad boxy bodies. *Phyllium cayabyabi* sp. nov. is most similar to *Phyllium arthurchungi* and the only feature we have found that might differentiate them is the abdominal shape, with *Phyllium arthurchungi* having a wider segment VI, and a slight undulation to segment VII, vs *Phyllium cayabyabi* sp. nov. which has a slightly narrower abdomen and segment VII with straight margins. *Phyllium cayabyabi* sp. nov. can be differentiated from *Phyllium cummingi* and *Phyllium gantungense* by abdominal segment V, which is *Phyllium cayabyabi* sp. nov. has widening margins, but in the other two species the margins are parallel.

**Figure 8.**
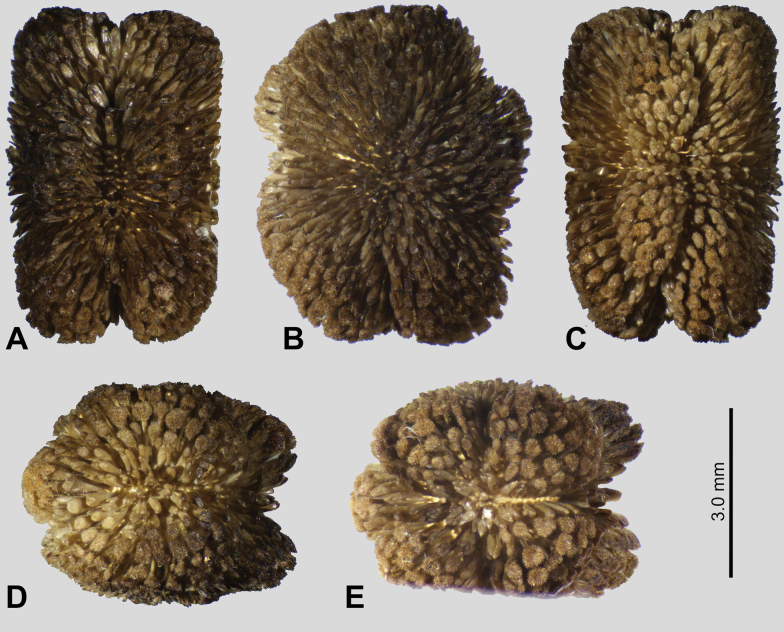
*Phyllium cayabyabi* sp. nov. paratype egg (Coll RC #20-138). A. Dorsal view; B. Lateral view (dorsum to the left); C. Ventral view; D. Opercular (anterior) view; E. Posterior view. Scale bar: 3.0 mm (A–E).

**Figure 9.**
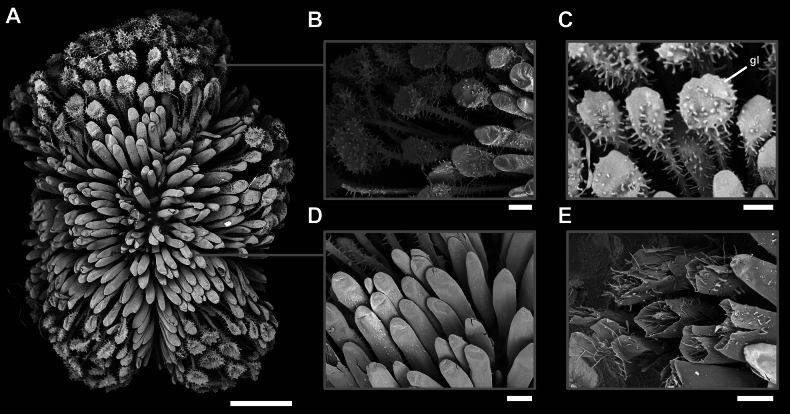
*Phyllium cayabyabi* sp. nov. paratype egg (Coll RC #20-140). Scanning electron micrographs of the egg. A Overview of the egg, lateral view. B–E. Details of the egg surface structures; B. Spatulate pinnae; C. Spatulate tip with glue; D. Hollow columnar pinnae; E. Cross-sections of removed pinnae showing fibrillar infill. Abbreviations: gl, glue residuals. Scale bars: 1 mm (A), 100 µm (B–E).

Eggs of *Phyllium cayabyabi* sp. nov. (Fig. 8) are unlike any known phylliid species egg due to the autapomorphic traits of spatulate pinnae with short chorionic outgrowths (Fig. 9B) and hollow, columnar pinnae densely covering the entire egg surface (Fig. 9D). While the above discussed species males and females all look very similar, only the eggs of *Phyllium cayabyabi* sp. nov. allow for reliable morphological differentiation. To contrast, the eggs of closely related species *Phyllium gantungense* and *Phyllium arthurchungi* (Fig. 1) both have large pits along their lateral surfaces arranged in a 2 × 4 pattern, completely unlike *Phyllium cayabyabi* sp. nov. eggs.

##### Description.

**Female. *Coloration*.** Coloration description is based upon photos of the type material shared with the authors of the live specimens prior to preservation. The general coloration is pale green throughout. The only areas that differ are the antennae that are somewhat orange/tan and some of the more prominent veins of the tegmina which are brown.

***Morphology*. *Head*** capsule longer than wide, with a vertex that is somewhat roughly textured, and marked with minimal granulation along the posterior (Fig. 6A). The posteromedial tubercle is present, singularly lobed, but not very prominent (Fig. 6A). Frontal convexity is broad and ending in a blunted point; there are several short setae across the surface. Compound eyes slightly protruding from the head capsule, not bulbous, taking up ~ ¼ of the head capsule lateral margins (Fig. 6A). Ocelli absent. Antennal fields slightly wider than the first antennomere width.

**Figure 10.**
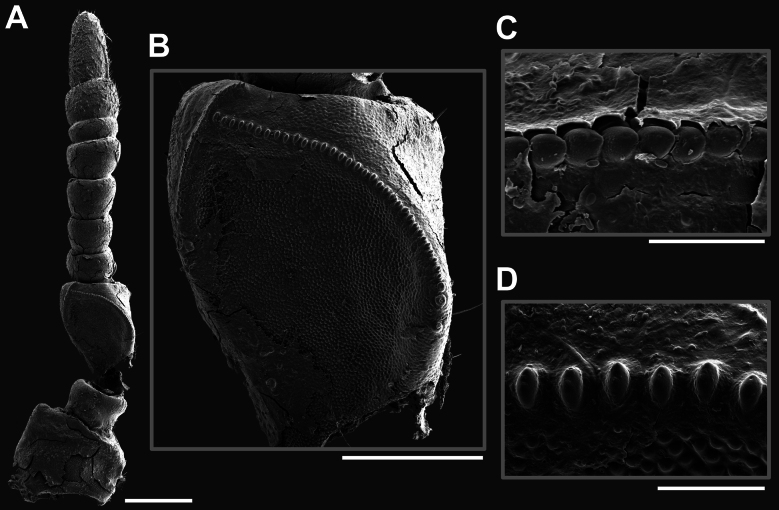
*Phyllium cayabyabi* sp. nov. holotype female antenna and fine details (Coll RC #20-135). Scanning electron micrographs of female antenna. A. Overview of the antenna, medial view; B. Third antennomere; C. Stridulatory ridge; D. Stridulatory file. Scale bars: 500 µm (A), 300 µm (B), 30 µm (B).

***Antennae*** consist of ten segments, with the terminal segment approx. the same length as the preceding 2½ segments’ lengths combined (Fig. 10A). Antennomeres I–VIII are smooth, and sparsely marked with short setae, the terminal two antennomeres are covered in short, dense setae, giving these segments a fuzzy appearance (Fig. 10A). Stridulatory file of antennomere III has 33–35 teeth, and the stridulatory ridge has 33 or 34 teeth (Fig. 10B).

***Thorax*.** Pronotum with slightly concave anterior margin and lateral margins that anteriorly start wide, angle inward strongly to the posterior margin which is ~ ½ the width of the anterior margin (Fig. 6A). The pronotum anterior margin and the lateral margins have prominent rims, while the posterior margin is less prominent. The pronotum surface is relatively smooth, with only a prominent pit in the center and a distinct furrow anterior to the center (Fig. 6A). Prosternum and the anterior 1/3 of the mesosternum are covered with irregularly spaced granulation; the remainder of the mesosternum and the metasternum are slightly wrinkled but lack nodes. Mesoprescutum slightly longer than wide, lateral rims with six or seven small tubercles (Fig. 6E). Mesoprescutum anterior rim prominently raised into a distinct and finely pointed sagittal spine (Fig. 6C). Mesoprescutum surface slightly lumpy and only slightly raised along the sagittal crest, which has several haphazardly located nodes along its length, not perfectly aligned along the plane (Fig. 6E). Mesopleurae narrow for the anterior 1/3 until they begin to diverge and angle prominently away with nearly straight margins (Fig. 6E). Mesopleuron lateral margin with six or seven medium sized nodes, mostly situated on the anterior 1/2, and the posterior 1/2 only has some minimal granulation (Fig. 6E). Face of the mesopleuron slightly wrinkled (Fig. 6E).

***Wings*.** Tegmina long, reaching the anterior margin of abdominal segment VIII. Tegmina venation; the subcosta (Sc) is the first vein in the forewing, running parallel with the margin for the first 1/2, and then bending and running towards the margin. The subcosta runs for ~¼ of the tegmina length. The radius (R) spans the central portion of the forewing with two subparallel (slightly diverging) branched veins; the first radius (R1) branches ~¼ of the way through the wing length and terminates ~1/3 of the way through the wing length; the radial sector (Rs) branches ~1/3 of the way through the wing length and terminates near the distal 1/3 of the wing length. There is a weak continuation of the radius following the prominent Rs branching which continues on as a short but distinct R–M crossvein that weakly connects the two veins. The media (M) is bifurcate with both the media anterior (MA) and media posterior (MP) terminating near to the posterior of the tegmina. The cubitus (Cu) is also bifurcate, branching near the posterior ¼ of the wing into the cubitus anterior (CuA) and cubitus posterior (CuP) which both terminate near the wing apex. The first anal vein (1A) is simple and fuses with the cubitus ~¼ of the way through the tegmina length. Alae vestigial, only small nubs.

***Abdomen*.** Abdominal segments II through the anterior ½ of IV gradually and uniformly diverging. The posterior ½ of segment IV through the anterior ½ of segment VII are only slightly diverging to the widest point of the abdomen. Abdominal segment VII is rounded ca 90 degrees with posterior margins angled almost directly inward where they meet the notably narrower segment VIII. Segments VIII–X have straight, converging margins ending in a broad rounded apex (Fig. 6G).

***Genitalia*.** Subgenital plate starts at the anterior margin of tergum VIII, is moderately broad, and extends ¾ of the way onto tergum X. The shape is somewhat tiered into approximately three widths as it converges to a finely pointed apex (Fig. 6F). Gonapophyses VIII are long and not particularly broad, exceeding the apex of the abdominal tergum X slightly; gonapophyses IX are obstructed from view (Fig. 6F). Cerci flat, slightly broadening to the apical 1/3 into a somewhat blade-like end, with a slightly granular surface (Fig. 6F).

***Legs*.** Profemoral exterior lobe broad and arching end to end, with a width slightly narrower than the width of the interior lobe (Fig. 6A). Margin of the profemoral exterior lobe slightly granular (Fig. 6A). Profemoral interior lobe slightly more than 2 × as wide as the greatest width of the profemoral shaft, approximately right angled, and marked with four large, triangular teeth with looping gaps between them, arranged in a two-wide gap-two pattern (Fig. 6A). Mesofemoral interior and exterior lobes approx. as wide as the mesofemoral shaft width. Mesofemoral exterior lobe with two small, distally pointing teeth on the distal ½ of the lobe with a wide gap between them. Mesofemoral exterior lobe is somewhat angled, not as smoothly arching as in the interior lobe. Mesofemoral interior lobe with six small, distally pointing teeth on the distal 2/3 of the lobe, with the teeth somewhat arranged into pairs. Metafemoral interior lobe arcs end to end, with the proximal 1/3 notably thinner and smooth and slightly widening out to the distal 2/3 which is wider and armed with seven or eight dulled, small teeth. Metafemoral exterior lobe lacks dentation, is thinner than the shaft width, and runs parallel with the shaft throughout its length. Protibia exterior lacking a lobe (Fig. 6A). Protibiae interior lobe spans the entire length of the protibiae (although the distal end is very thin and not prominent) and is ~1.5 × the width of the protibiae shaft itself. The lobe is roundly triangular with the widest portion near the middle, and the proximal end more distinct than the distal end. Mesotibiae and metatibiae simple, lacking exterior and interior lobes.

***Measurements*** (mm). Holotype, female: body length (including cerci and head, excluding antennae): 85.9, length/width of head: 8.3/6.8, antennae: 4.4, pronotum: 5.9, mesonotum: 8.2, length of tegmina: 54.0, greatest width of abdomen: 36.7, profemora: 14.9, mesofemora: 13.5, metafemora: 17.1, protibia: 9.8, mesotibia: 10.5, metatibia: 15.0.

**Male. *Coloration*.** Coloration based upon the dead, dried type specimen which is somewhat discolored (Fig. 7). Overall coloration pale green/yellow throughout. The compound eyes are brownish-red, and the antennae are darker/gray. Protibia interior lobe and margin of the profemoral interior lobe with several brown/tan markings

***Morphology*. *Head*** capsule slightly longer than wide, with a vertex that is slightly lumpy and marked throughout with a few small, widely distributed nodes (Fig. 7A). The posteromedial tubercle is singularly pointed, small, and not notably raised above the head capsule (Fig. 7A, F). Frontal convexities stout and bluntly pointed with a few sparse setae. Compound eyes large and bulbous, occupying ~2/5 of the head capsule lateral margins (Fig. 7A). There are three distinct ocelli raised above the capsule and located between the compound eyes (Fig. 7A).

***Antennae*** (including the scapus and pedicellus) consist of 25 segments (Fig. 7A), all segments except the scapus and pedicellus and terminal five segments are covered in numerous setae where most are as long as the antenna segment is wide. The terminal five segments are covered in dense, short setae and the scapus and pedicellus are nearly completely bare with only a few sparse setae.

***Thorax*.** Pronotum with slightly convex anterior margin and straight lateral and posterior margins. Posterior margin is ~½ the width of the anterior margin. The anterior margin is well-developed, the lateral margins are moderately formed, and the posterior margin is weakly formed (Fig. 7D). Face of the pronotum is marked by a distinct pit in the center, a sagittal furrow on the anterior ½, and slight perpendicular furrows originating from the central pit. The pronotum surface is only slightly lumpy but lacking distinct granulation (Fig. 7D). Prosternum surface is lumpy with small nodes. Mesosternum surface anterior 1/3 marked heavily with granulation, the remainder of the mesosternum surface is wrinkled but lacks notable nodes. Metasternum surface mostly wrinkled throughout, and the anterior margin central area is additionally marked with granulation. Mesoprescutum longer than wide, with lateral margins that are slightly converging to the posterior margin which is only slightly narrower than the anterior margin (Fig. 7D). Lateral margins of the mesoprescutum with five or six moderately formed tubercles of a somewhat uniform size (Fig. 7D). Mesoprescutum surface wrinkled and slightly raised along the sagittal plane which is marked with four or five distinct nodes and the remainder of the surface has slight granulation (Fig. 7D, F). Mesoprescutum anterior rim moderately formed with a distinct sagittal spine, and the remainder of the rim surface is slightly wrinkled (Fig. 7D, F). Mesopleurae begin on the anterior mesoprescutum margin, begin very narrow, and diverge slowly at a gradually increasing angle from the anterior to the posterior but are never notably wide throughout their length (Fig. 7D). Mesopleuron lateral margin with four or five small tubercles and a few small nodes interspersed throughout the length except for the posterior 1/3 of the margin which is relatively smooth (Fig. 7D). Mesopleuron face moderately wrinkled and marked by a distinct pit near the center.

***Wings*.** Tegmina moderate length, extending ¾ of the way onto abdominal segment IV. Tegmina wing venation: the subcosta (Sc) is the first vein, is simple, and terminates ~1/3 of the way through the overall wing length. The radius (R) spans nearly the entire length of the tegmina with the first radius (R1) branching ~1/3 of the way through the tegmina length and terminates on the margin slightly less than ½ through the length. There is also a second radius (R2) that originates near the middle, and a third radius (R3) which originates ~3/5 of the way through the wing length. The radial sector runs towards the wing apex, but near the terminal 1/3 angles towards the margin, slightly away from the apex. The media (M) spans the entire length of the tegmina running side by side along the radius/radial sector for most of the length, with a first media posterior (MP1) branching off near the midlength of the tegmina and running angled towards the cubitus, a second media posterior (MP2) branches off ~3/5 of the way through the length, and the media anterior (MA) runs straight to the tegmina apex. The cubitus (Cu) cuts across the tegmina to the margin ~1/3 of the way through the length and runs along the edge of the tegmina where the media posterior veins fuse with it and as the cubitus reaches the apex it fades. The first anal (1A) vein terminates upon reaching the cubitus ~1/3 of the way through the tegmina length. Alae well-developed in an oval fan configuration, long, reaching to the middle of abdominal segments VIII. Ala wing venation hidden from view due to the alae being folded in the type specimen.

***Abdomen*.** Lateral margins of abdominal segment II parallel, III diverging slightly, IV diverging at a more prominent angle for the anterior 2/3 and then slightly less strongly for the posterior 1/3, V slightly diverging, VI diverges slightly for the anterior 2/3 to the widest point of the abdomen, then converges on the posterior 1/3, followed by all preceding segments converging strongly at first and then more gradually towards the apex (segment X), which is broad and rounded.

***Genitalia*.** Poculum broad and ends with a flat, blunted apex that slightly passes the anterior margin of abdominal segment X with a margin that is nearly straight (Fig. 7E). Cerci long, slender, and nearly flat, with subparallel margins, with ~2/3 of their length extending from under abdominal segment X. The cerci surfaces are slightly granular and there are numerous short setae along the margins (Fig. 7E). Vomer broad and stout with straight sides evenly converging to the apical hook which is thick and has a singular point (Fig. 7E).

***Legs*.** The profemoral exterior lobe arcs end to end and is narrow, approx. the same width as the profemoral shaft at its widest. The profemoral exterior lobe margin is slightly granular (Fig. 7A). The profemoral interior lobe is obtusely triangular and at its greatest width it is ~2 × the greatest width of the profemoral shaft. The profemoral interior lobe is ornamented on the distal ½ with four serrate teeth arranged as small tooth-large tooth-wide gap-large tooth-small tooth (Fig. 7A). Mesofemoral exterior lobe and interior lobe are of similar shapes and widths, both arching end to end but are slightly wider on the distal 2/3. Both lobes at their widest are approx. as wide as the mesofemoral shaft width. The only notable difference between these two lobes is that the interior lobe has five small teeth on the distal 1/2. The mesofemoral exterior lobe is unornamented. Metafemoral exterior lobe lacks dentition and has a straight, thin margin along the metafemoral shaft. Metafemoral interior lobe is approx. as wide as the metafemoral shaft width, arcs end to end, but is thinner on the proximal 1/3, the distal 1/3 is marked with six small serrate teeth. Protibiae lacking exterior lobe, interior lobe mostly situated in the middle of the shaft with the distal end without lobe and the proximal end very thin. The protibial interior lobe is a small, rounded triangle with the widest portion just distal to the midlength (Fig. 7A). Meso- and metatibiae simple, lacking lobes completely.

***Measurements of paratype male* [mm].** Length of body (including cerci and head, excluding antennae) 65.4, length/width of head 4.4/3.8, antenna 26.2, pronotum 3.5, mesonotum 4.9, length of tegmina 24.5, length of alae 42.4, greatest width of abdomen 20.2, profemora 10.4, mesofemora 10.0, metafemora 11.0, protibiae 6.5, mesotibiae 7.2, metatibiae 10.8.

**Eggs** (Figs 8, 9). The overall egg shape is difficult to discern fully due to the long and dense pinnae, but appears to be somewhat reniform, with the ventral surface protruding slightly and the dorsal surface slightly curved inward. The entire egg capsule is covered by spatulate pinnae with short chorionic outgrowths (Fig. 9B) and hollow, columnar pinnae densely covering the entire egg surface (Fig. 9D). These two types are somewhat interspersed, but the roughly textured spatulate pinnae are more prominent along the anterior and posterior margins, while the smoother columnar pinnae are more prominent on the flat surfaces. The operculum is nearly circular and rimmed by the roughly textured spatulate pinnae which are slightly shorter than the pinnae on the remainder of the egg capsule. The micropylar plate is mostly obstructed from view but appears to be thin and only situated in the middle of the egg capsule. The capsule surface below the dense pinnae appears to be rather smooth and paler in color than the dark brown pinnae covering the surface.

##### Measurements including the extended pinnae [mm].

 Length (including operculum expansion): 6.0–6.6; maximum width of capsule when viewed from lateral aspect 4.8–5.5; length of micropylar plate 2.8–3.4.

##### Etymology.

Patronym; named to honor Victor Cayabyab, a collaborator of the Montreal Insectarium for the last 30 years and a very good friend to the fourth author.

##### Distribution.

At present only known from the type locality of Malinau, in North Kalimantan, Indonesia (Fig. 2).

#### ﻿ Phyllium crapulatum

Taxon classificationAnimaliaPhasmatodeaPhylliidae

Cumming, Foley, Hennemann, Le Tirant & Büscher sp. nov.

ECA53CA0-D5FC-586F-8145-88BAAB6AB0B6

https://zoobank.org/5DC41DD0-BF37-4984-8D10-46F0765547BA

Fig. 11

##### Type material.

***Holotype*** (♂): Indonesia • West Kalimantan, Mount Bawang, 00 53.5'N, 109 22.2'E, Elv. 245 Meters, May, 2023. Tissue sample: SLT069 [IMQC].

##### Differentiation.

**Figure 11.**
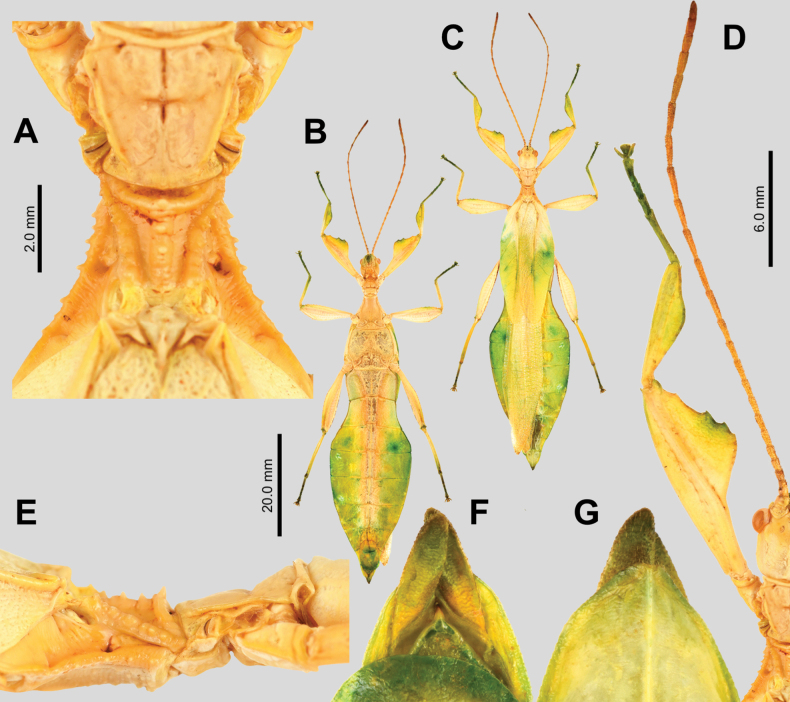
*Phyllium crapulatum* sp. nov. holotype (IMQC). A. Details of thorax, dorsal; B. Habitus, ventral; C. Habitus, dorsal; D. Details of antenna and front leg, dorsal; E. Posterior of head through thorax, lateral (head to the right); F. Terminalia ventral; G. Terminalia dorsal. Scale bar: 20.0 mm (B, C).

Female, egg, and freshly hatched nymph unknown. Male *Phyllium crapulatum* sp. nov. are most similar to *Phyllium rubrum* and *Phyllium bradleri* due to their similar sizes, abdominal shapes, tegmina lengths, and thorax shape and spination. *Phyllium crapulatum* sp. nov. appears most similar to *Phyllium rubrum* due to the same length tegmina, similar profemoral exterior lobe width, and similar thorax shape and spination. These species were recovered as closely related in our genetic phylogeny (Fig. 1) so their morphological similarity is not surprising. Only subtle morphological differences could be found between these species. In *Phyllium rubrum*, the males have prominently colored ventral surfaces of their coxae (red; a unique occurrence as typically bright coxae colors are only found in females), vs *Phyllium crapulatum* sp. nov. which does not appear to have colored coxae. Hopefully, once females and eggs can be observed of this species, more morphological differences can be illustrated. *Phyllium crapulatum* sp. nov. can be differentiated from *Phyllium bradleri* by the protibial interior lobe shape, as *Phyllium bradleri* has the distal end of the rounded triangle lobe notably thinner than the proximal end of the lobe, vs *Phyllium crapulatum* sp. nov. which has a interior lobe that is more evenly distributed across the length, with a distal end similar in width to the proximal end width (Fig. 11D).

##### Description.

**Male. *Coloration*.** Coloration based upon the dead, dried holotype (Fig. 11). Overall coloration pale green and yellow throughout with highlights of tan/orange. The antennae and compound eyes are the darkest areas on the specimen with burnt orange color. Abdominal segment V has a pair of slightly lighter eye spots that are not prominent. Ventral coxae coloration matches the surrounding tissue.

***Morphology*. *Head*** capsule slightly longer than wide, with a vertex that is rather smooth. The posteromedial tubercle is singularly pointed and distinctly raised above the head capsule (Fig. 11E). Frontal convexities stout and bluntly pointed with sparse setae. Compound eyes large and bulbous, occupying ~2/5 of the head capsule lateral margins (Fig. 11D). There are three well-developed ocelli distinctly raised above the capsule and located between the compound eyes.

***Antennae*** (including the scapus and pedicellus) consist of 25 segments, all segments except the scapus and pedicellus and terminal five segments are covered in dense setae where most are as long as the antenna segment is wide (Fig. 11D). The terminal five segments are covered in dense short setae and the scapus and pedicellus are nearly completely bare with only a few sparse setae.

***Thorax*.** Pronotum with slightly concave anterior margin and straight lateral margins that converge to a slightly convex posterior margin that is ~½ the width of the anterior margin (Fig. 11A). Anterior and lateral margins of the pronotum have distinct rims and the posterior margin has a weakly formed rim (Fig. 11A). Face of the pronotum is marked by a distinct pit in the center, a sagittal furrow on the anterior ½, and slight perpendicular furrows originating from the central pit. The pronotum surface is slightly lumpy and lacking distinct granulation (Fig. 11A). Prosternum surface is slightly granular. Mesosternum surface anterior 1/3 slightly granular, the remainder of the mesosternum surface is relatively smooth (Fig. 11B). Metasternum surface finely wrinkled throughout. Mesoprescutum longer than wide, with lateral margins that slightly converge to the posterior margin which is ~¾ as wide as the anterior margin (Fig. 11A). Lateral margins of the mesoprescutum with six or seven stout tubercles with the anterior three the most prominent (Fig. 11A). Mesoprescutum surface slightly raised along the sagittal plane and is marked with three or four distinct but small spines and the remainder of the surface is relatively smooth (Fig. 11A). Mesoprescutum anterior rim moderately formed with a distinct sagittal spine, and the remainder of the rim surface is relatively smooth (Fig. 11E). Mesopleurae begin on the anterior mesoprescutum margin and diverge at a gradually increasing angle from the anterior to the posterior but are relatively narrow throughout their length (Fig. 11A). Mesopleuron lateral margin with seven or eight moderately formed tubercles and a few small nodes interspersed throughout the length (Fig. 11A). Mesopleuron face moderately wrinkled and marked by a distinct pit on the anterior 1/3 and a smaller pit on the posterior 1/3.

***Wings*.** Tegmina moderate length, extending ¼ of the way onto abdominal segment IV. Tegmina wing venation: the subcosta (Sc) is the first vein, is simple, and terminates ~1/3 of the way through the overall wing length. The radius (R) spans the entire length of the tegmina with the first radius (R1) branching ~1/3 of the way through the wing length and terminating at the wing margin ~½ of the way through the wing length. There is an additional radius (R2) branching near the midlength of the tegmina and the radial sector runs straight to the wing apex. The media (M) also spans the entire length of the tegmina and runs side by side along the radius/radial sector with the first media posterior (MP1) branching off near the tegmina midlength, followed by a second media posterior (MP2) near the distal 2/5, and the media anterior (MA) runs straight to the tegmina apex. The cubitus (Cu) cuts across the tegmina to the margin ~1/3 of the way through the length and runs along the edge of the wing where the first and second media posterior veins fuse with it and as the cubitus reaches the apex it fades. The first anal (1A) vein terminates upon reaching the cubitus 1/3 of the way through the tegmina length. Alae well-developed in an oval fan configuration, long, reaching to the posterior of abdominal segments IX. Ala wing venation not visible in the holotype specimen.

***Abdomen*.** Lateral margins of abdominal segment II slightly converging, III diverging slightly, IV diverging to the widest point 2/3 of the way through the segment length and the remaining 1/3 of the segment is parallel, V parallel sided or slightly subparallel (converging slightly), VI through X converging gradually with smooth margins, giving the abdomen a spade-shaped appearance.

***Genitalia*.** Poculum broad and ends in an apex that slightly passes the anterior margin of the abdominal segment X with a margin that is broad and straight (Fig. 11F). Cerci long and slender, with slightly > ½ of their length extending from under abdominal segment X, nearly flat, covered in a granulose surface and several short setae with those along the margins slightly longer (Fig. 11F). Vomer broad and stout with straight sides evenly converging until near the apex where the margins converge more sharply to the apical hook which is thick and has a singular point (Fig. 11F).

***Legs*.** The profemoral exterior lobe is narrow, approx. the same width as the profemoral shaft at its widest. The profemoral exterior lobe margin is relatively smooth (Fig. 11D). The profemoral interior lobe is obtusely triangular and at its greatest width it is ~2 × the greatest width of the profemoral shaft. The profemoral interior lobe is ornamented with four serrate teeth arranged in a three-one pattern with looping gaps between them, where the third and fourth tooth has a notable wider gap between them (Fig. 11D). The central two teeth are notably larger than the first and fourth teeth (Fig. 11D). Mesofemoral exterior lobe arcs end to end but is slightly wider on the distal 1/3 which is marked with two teeth, while the proximal 2/3 of the lobe is thinner and lacks teeth. Mesofemoral interior lobe and mesofemoral shaft are approximately the same width, and the exterior lobe is slightly thicker. The mesofemoral interior lobe is slightly broader on the distal end and the distal ½ is ornamented with six small serrate teeth while the proximal portion of the lobe is thin and lacks teeth. Metafemoral exterior lobe has a straight margin along the metafemoral shaft and is mostly unornamented but does have two small teeth on the distal 1/3. Metafemoral interior lobe arcs end to end with nine or ten sharply serrate teeth on the distal ½, which is slightly wider than the smooth proximal portion of the lobe. Protibia lacking exterior lobe; interior lobe reaching end to end as a rounded triangle with the widest portion slightly distal to the midlength with a maximum width of ~1.5 × as wide as the protibial shaft width (Fig. 11D). Meso- and metatibiae simple, lacking lobes completely.

***Measurements of holotype male* [mm].** Length of body (including cerci and head, excluding antennae) 63.8, length/width of head 4.4/3.8, antennae 28.4, pronotum 3.4, mesonotum 4.9, length of tegmina 25.0, greatest width of abdomen 16.3, profemora 10.9, mesofemora 10.3, metafemora 12.8, protibiae 6.5, mesotibiae 6.6, metatibiae 9.8.

##### Etymology.

Latin in origin, adjective. Meaning drunk or intoxicated. For anyone who has seen living phylliids, when in the wild on a bush or in a tree, they blend completely with the leaves swaying in the breeze. However, most people rarely see them in the wild, more often they are seen handled as pets indoors or in terrariums which lack a gusty breeze. This results in the leaf insect swaying wildly, as is their natural predisposition (Leigh 1912; Steiniger 1933), but looking very much like a drunken leaf stumbling home after a wild evening of too much merriment. Hence, the binomial meaning “drunken leaf”.

##### Distribution.

At present known from the type locality; Mount Bawang in West Kalimantan, Indonesia (Fig. 2).

##### Remarks.

This species is part of a small clade comprised of *Phyllium rubrum* (which is found on Peninsular Malaysia; Fig. 1) and an unidentified nymph sample (*Phyllium* sp. 5 “Mt. Bawang”) which was included within Bank et al. (2021b) from the same locality as *Phyllium crapulatum* sp. nov., Mount Bawang, West Kalimantan. Phylogenetically, this unidentified nymph “*Phyllium* sp. 5” was recovered as more closely related to *Phyllium rubrum* vs *Phyllium crapulatum* sp. nov., suggesting that there are likely two different *Phyllium* species found on Mount Bawang. Unfortunately, as the specimen from Bank et al. (2021b) is a small nymph, a morphological comparison between *Phyllium crapulatum* sp. nov. and this nymph cannot be made, and the nymph must remain unidentified.

#### ﻿ Phyllium hennemanni

Taxon classificationAnimaliaPhasmatodeaPhylliidae

Cumming, Foley, Le Tirant & Büscher sp. nov.

371848E3-7B98-5710-B15D-86D1AEBA6D41

https://zoobank.org/C2124DAB-6E57-41E2-8B69-821E809B1E72

Figs 12, 13, 14

##### Type material.

***Holotype*** (♀): South Sulawesi, Sulawesi Selatan, Bungadidi, II.2011, local collector, ex coll. Sigetake Suzuki; tissue sample SB0642; [ZSM]. ***Paratypes***: (2♀♀, 18 eggs): (1♀) South-East Sulawesi, Sulawesi Tenggara, Tiulapolu (Tipulu), III.2008, local collector coll. Sigetake Suzuki. (1♀) South-East Sulawesi, Sulawesi Tenggara, Tiulapolu (Tipulu), III.2008, local collector ex coll. Sigetake Suzuki [coll. FH, No. 1091-1]. (5 eggs) South-East Sulawesi, Sulawesi Tenggara, Tiulapolu (Tipulu), III.2008, local collector ex coll. Sigetake Suzuki [coll. FH, No. 1091-E1]. (1 egg) [ex ovipositor HT]: South Sulawesi, Sulawesi Selatan, Bungadidi, II.2011, local collector, ex coll. Sigetake Suzuki [ZSM]. (9 eggs): South Sulawesi, Sulawesi Selatan, Bungadidi, II.2011, local collector, ex coll. Sigetake Suzuki [coll. FH, No. 1091-E2]. (3 eggs): South Sulawesi, Sulawesi Selatan, Bungadidi, II.2011, local collector, ex coll. Sigetake Suzuki [Coll RC, 20-086, 20-087, and 20-088].

##### Differentiation.

Male unknown. Female *Phyllium hennemanni* sp. nov. (Fig. 13) are most similar to *Phyllium mamasaense* and *Phyllium letiranti* due to similar femoral lobe shapes/serration and genitalia. *Phyllium hennemanni* sp. nov. can be differentiated from *Phyllium letiranti* by the ventral coxae coloration, as *Phyllium letiranti* has orange ventral coxae coloration while *Phyllium hennemanni* sp. nov. has a distinct black spot on both the meta- and mesocoxae. Additionally, these species can be differentiated by the mesopleurae of the thorax as *Phyllium hennemanni* sp. nov. has small nodes throughout the length (giving them a rough marginal texture; Fig. 13F) and the mesopleurae angle towards the anterior at a stronger angle, terminating before reaching the anterior end of the mesoprescutum (vs *Phyllium letiranti* which has distinct spiniform tubercles and the mesopleurae reaching to the anterior margin of the mesoprescutum). *Phyllium hennemanni* sp. nov. females are most morphologically similar to *Phyllium mamasaense* and even have the same ventral coxae coloration (black spots on both the meta- and mesocoxae), but these species can be differentiated by their thorax and antennae morphology. In *Phyllium hennemanni* sp. nov. the mesopleurae margins have small nodes (Fig. 13F) vs *Phyllium mamasaense* which has distinct spiniform tubercles. The number of antennomeres allow differentiation as *Phyllium mamasaense* has nine segments while *Phyllium hennemanni* sp. nov. has ten segments (due to the segment prior to the apical segment being split by a prominent suture and these two portions having different textures). The apical antennomere of *Phyllium mamasaense* is also stouter than the longer and thinner apical antennomere of *Phyllium hennemanni* sp. nov. (Fig. 13B).

**Figure 12.**
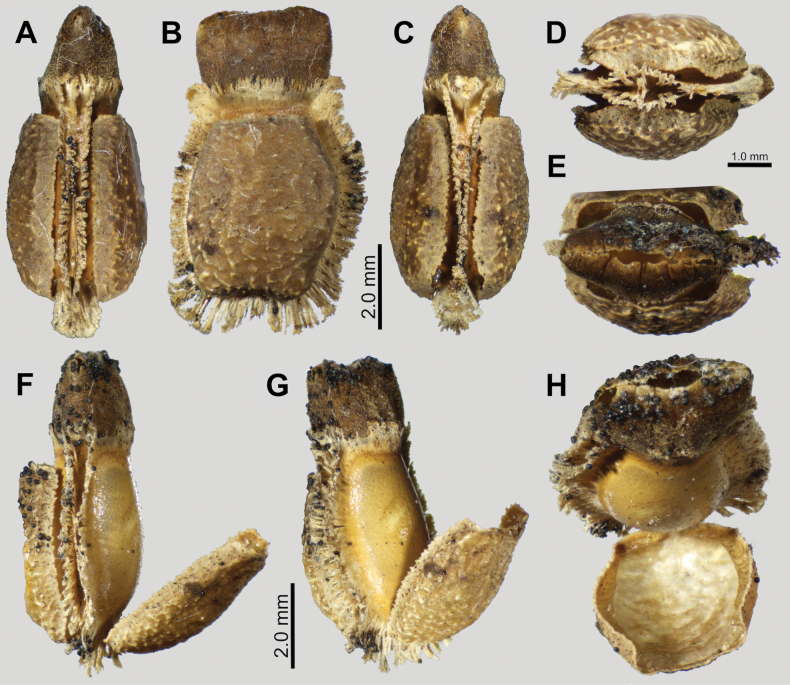
*Phyllium hennemanni* sp. nov. paratype eggs. A, B, C [Coll RC 20-086], D, E [Coll RC 20-087], F, G, H [Coll RC 20-088]. A. Dorsal view; B. Lateral view (dorsum to the left); C. Ventral view; D. Posterior view; E. Opercular (anterior) view; F. Dorsal view with lateral flap opened to expose egg capsule; G. Dorso-lateral view with lateral flap opened to expose egg capsule; H. Dorsal view with lateral flap opened to expose egg capsule and expose inner surface of the lateral flap. Scale bar: 2.0 mm (A, B, C, F, G); 1.0 mm (D, E, H).

Eggs of *Phyllium hennemanni* sp. nov. (Figs 12, 14) are distinct from all known phylliid species eggs due to the autapomorphic trait of a pair of posteriorly attached, lateral flaps which create a hollow cavity between the flap and the actual egg capsule (Fig. 12). The main feature of *Phyllium hennemanni* sp. nov. eggs which is similar to other known phylliid species is the raised, fused frill running around the margin of the operculum (Fig. 12A). The only other species with such a distinct fused capitular frill are *Phyllium mamasaense*, *Phyllium ericoriai*, and *Phyllium bonifacioi*. While the opercular coverage of *Phyllium ericoriai* and *Phyllium bonifacioi* is formed by a frill of upright standing pinnae, the structure in *Phyllium mamasaense* and *Phyllium hennemanni* sp. nov., are more similar as these opercular pinnae are fused on their entire length (Fig. 14C). All three of these species besides *Phyllium hennemanni* sp. nov. lack lateral flaps on the egg capsule and instead have reinforced ribs (pinnae “type 4” as designated in Büscher et al. 2023). These three species can be differentiated from *Phyllium hennemanni* sp. nov. by these lateral reinforced ribs (and these reinforced ribs allow differentiation from all other phylliids as well).

**Figure 13.**
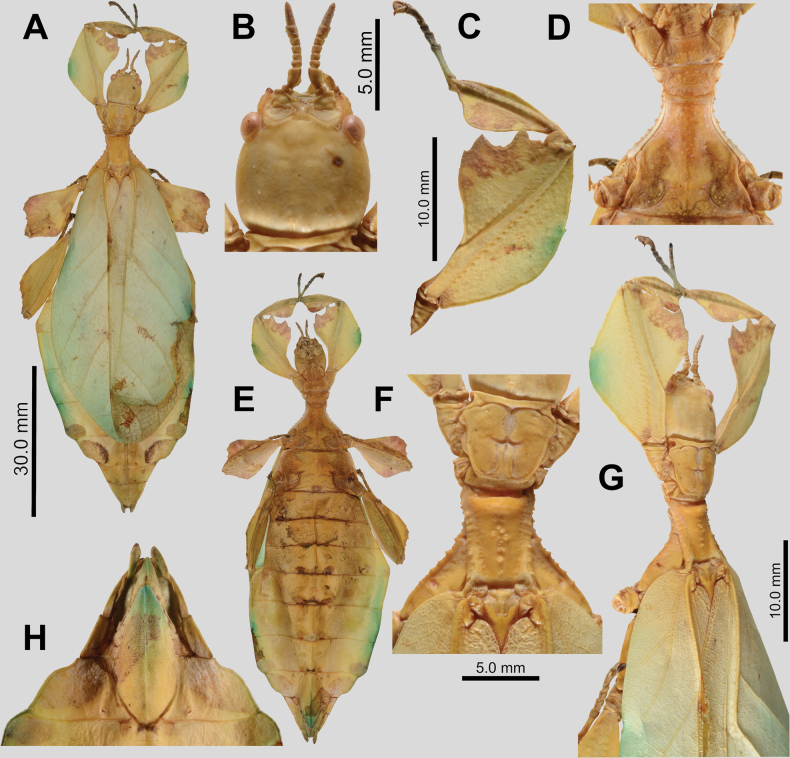
*Phyllium hennemanni* sp. nov. holotype female (ZSM). A. Habitus, dorsal; B. Detail of head and antennae, dorsal (5.0 mm scale bar to the right); C. Front right leg, dorsal; 10.0 mm scale bar; D. Thorax, ventral; E. Terminalia, ventral; F. Habitus, ventral; G. Details of the thorax, dorsal (5.0 mm scale bar below); H. Dorsolateral view of the front legs, head, and thorax; 10.0 mm scale bar. Scale bar 30.0 mm (A, E).

**Figure 14.**
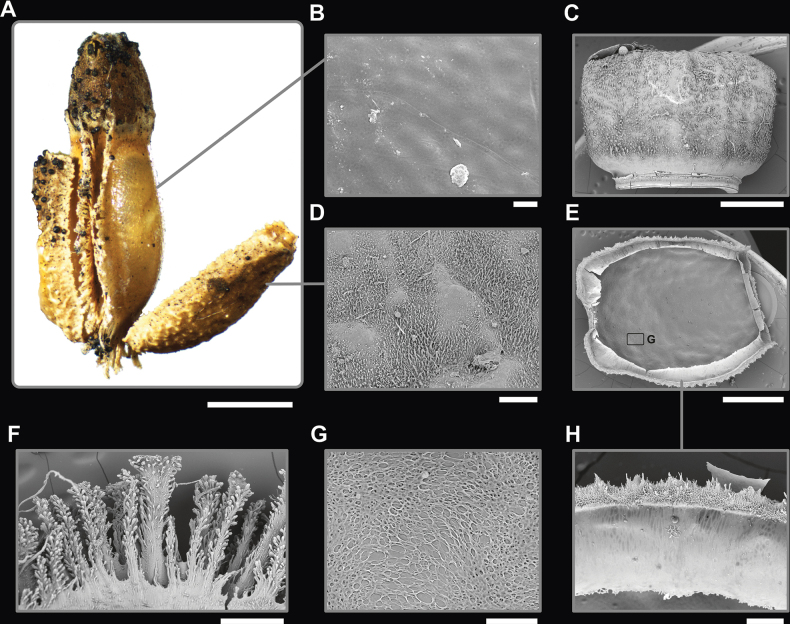
*Phyllium hennemanni* sp. nov., scanning electron micrographs of a paratype egg [Coll RC 20-088]. A Overview of the egg, dorsal view. B–H. Details of the egg morphology: B. Smooth interior surface: C. Operculum: D. Exterior surface of the lateral flap: E. Interior view of the lateral flap: F. Pinnae; G. Magnification of the inside of the lateral flap; H. Membranous rim of the lateral flap. Scale bars: 2 mm (A), 1 mm (C, E), 10 µm (B), 100 µm (D, G, H), 200 µm (F).

##### Description.

**Female. *Coloration*.** Coloration description is based upon the type material, which is dead and dried reasonably well (not too many dark rotten areas; Fig. 13). The general coloration is pale green throughout (although some areas have faded to yellow, likely due to the drying process). There are several areas, commonly occurring on other *Phyllium* species, which appear to be more variably marked with muddled brown/tan coloration; these areas are: the protibiae, profemoral interior lobe, mesofemoral lobes, and the margins of abdominal segments VII and VIII. Meso- and metacoxae ventrally marked with a dark spot.

***Morphology*. *Head*** capsule approximately as long as wide, with a vertex that is smooth, lacking granulation (Fig. 13B). The posteromedial tubercle is present, singularly lobed, but not very prominent (Fig. 13B). Frontal convexity broad and ending in a blunted point; there are several short setae across the surface. Compound eyes slightly protruding from the head capsule, not bulbous, taking up ~¼ of the head capsule lateral margins (Fig. 13B). Ocelli absent. Antennal fields slightly wider than the first antennomere width.

***Antennae*** consist of ten segments, with the terminal segment the narrowest and approx. the same length as the preceding four segments’ lengths combined (Fig. 13B). Antennomeres VIII and IX appear derived from a single segment as they are tightly situated, but have a prominent suture separating them into two, and each segment has a distinct texture, with segment IX a rough, fuzzy texture (like segment X) and segment VIII smoother with sparse setae (like on segment VII; Fig. 13B). Antennomeres I–VIII are smooth, and sparsely marked with short setae, the terminal two antennomeres are covered in short, dense setae, giving these segments a fuzzy appearance (Fig. 13B).

***Thorax*.** Pronotum with slightly concave anterior margin and lateral margins that anteriorly start wide, angle inward strongly, then for the middle portion are angled more gently, followed by a strong incurve to the posterior margin (Fig. 13F). The posterior margin is ~½ the width of the anterior margin (Fig. 13F). The pronotum anterior margin has a prominent rim, while the lateral and posterior margins are less prominent. The pronotum surface is relatively smooth, with only a prominent pit in the center and a few furrows (Fig. 13F). Prosternum, mesosternum, and metanotum are covered throughout by moderately spaced granulation (Fig. 13D). Mesoprescutum slightly longer than wide, lateral rims with eight or nine nodes (none particularly prominent; Fig. 13F). Mesoprescutum anterior rim prominently raised into a raised, broad sagittal spine (Fig. 13F). Mesoprescutum surface smooth except for the slightly raised mesoprescutum sagittal crest marked with variably granulation throughout the length, but only along the sagittal crest; areas lateral to the sagittal crest smooth (Fig. 13F). Mesopleurae begin to diverge ~1/3 of the way along the mesoprescutum, angle prominently away with straight margins (Fig. 13F). Mesopleurae lateral margins with six or seven nodes with interspersed granulation throughout, giving the margin a rough textured appearance (Fig. 13F). Face of the mesopleura slightly wrinkled, with two notable divots, one on the anterior margin and one near the middle (Fig. 13F).

***Wings*.** Tegmina long, reaching onto abdominal segment VIII. Tegmina venation; the subcosta (Sc) is the first vein in the forewing, running parallel with the margin for the first ½, and then bending and running towards the margin. The subcosta runs for ~1/3 of the tegmina length. The radius (R) spans the central portion of the forewing with two subparallel branched veins; the first radius (R1) branches ~¼ of the way through the wing length and terminates slightly proximal to the midline; the radial sector (Rs) branches ~1/3 of the way through the wing length and terminates near the distal 1/3 of the wing length. There is a weak continuation of the radius following the prominent Rs branching which continues on as a short but distinct R–M crossvein that weakly connects the two veins. The media (M) is bifurcate with both the media anterior (MA) and media posterior (MP) terminating near to the posterior of the tegmina. There is a weak continuation of the media following the prominent media posterior (MP) branching which continues on as a somewhat long M–Cu crossvein that fades before fully connecting the two veins. The cubitus (Cu) is also bifurcate, branching near the posterior ¼ of the wing into the cubitus anterior (CuA) and cubitus posterior (CuP) which both terminate near the wing apex. The first anal vein (1A) is simple and fuses with the cubitus ~ 1/3 of the way through the tegmina length. Alae vestigial, with their apex only just reaching abdominal segment I (~6.0 mm long as measured in a paratype).

***Abdomen*.** Abdominal segments II through the anterior 2/3 of IV gradually diverging. The posterior 1/3 of segment IV through the anterior 2/3 of segment VII are gradually and uniformly converging. The posterior 1/3 of segment VII is rounded inwards towards segment VIII which like VII starts converging gradually and then rounds inward to segment IX. Segments IX–X have straight, converging margins ending in a broad rounded apex (Fig. 13H).

***Genitalia*.** Subgenital plate starts at the anterior margin of tergum VIII, is moderately broad, and extends most of the way onto tergum X. The shape is approximately teardrop-shaped, with the apex a fine point (Fig. 3E). Gonapophyses VIII are long and moderately broad, reaching the apex of abdominal tergum X; gonapophyses IX are obstructed from view (Fig. 13H). Cerci flat, slightly broadening to the apical ¼, with a slightly granular surface (Fig. 13H).

***Legs*.** Profemoral exterior lobe broad, rounded, and obtusely angled, slightly narrower than the width of the interior lobe (Fig. 13C). Distal margin of the profemora with three small, finely pointed teeth (Fig. 13C). Profemoral interior lobe ~3 × as wide as the greatest width of the profemoral shaft, approximately right angled, and marked with three large teeth with looping gaps between them, each gap with a singular smaller tooth (Fig. 13C). Mesofemoral lobes significantly broadened on the distal 1/3, with the interior lobe greatest width ~2 × wider than the mesofemoral shaft width, and the exterior lobe greatest width ~1.5 × wider than the mesofemoral shaft width. Mesofemoral exterior lobe with three or four small, distally pointing teeth on the distal 1/3 of the lobe. Mesofemoral interior lobe with five small, distally pointing teeth on the distal 1/3 of the lobe. Metafemoral interior lobe arcs end to end, with the distal ½ slightly wider than the proximal ½ and marked with five or six serrate teeth on the distal ½ of the lobe. Metafemoral exterior lobe lacks dentation and has a width similar to the metafemoral shaft width. Protibiae exterior has a thin expansion near the middle, less than the width of the protibial shaft (Fig. 13C). Protibiae interior lobe spans the entire length of the protibiae and is ~2.5 × the width of the protibiae shaft itself. The lobe is roundly triangular with the widest portion on the distal ½. Mesotibiae and metatibiae simple, lacking exterior and interior lobes.

***Measurements of holotype female* [mm].** Length of body (including cerci and head, excluding antennae) 88.0, antennae 5.8, pronotum 5.9, mesonotum 10.5, length of tegmina 54.7, greatest width of abdomen 32.0, profemora 16.8, mesofemora 15.0, metafemora 18.5, protibiae 11.2, mesotibiae 10.3, metatibiae 15.0.

***Measurements of paratype females* [mm].** Length of body (including cerci and head, excluding antennae) 73.0–77.0, antennae 5.0–5.5, pronotum 5.2–5.5, mesonotum 9.0–9.5, metanotum 9.0, length of tegmina 46.0–50.0, length of alae 6.0, greatest width of abdomen 28.5–29.0, profemora 14.5–15.0, mesofemora 13.0–14.0, metafemora 15.0–16.0, protibiae 9.0–10.5, mesotibiae 9.0–9.5, metatibiae 13.0–14.5.

**Eggs** (Figs 12, 14). The lateral flaps and the fused capitular frill are brown; the feather-like pinnae are tan; and the exposed, smooth sides of the egg capsule under the lateral flaps are yellowish/tan in color. The actual egg capsule is notably smaller than the habitus due to the lateral flaps covering the entire lateral surfaces. These lateral flaps are connected to the capsule by a small, stiff section on the posterior of the egg. These lateral flaps are slightly convex and have an irregular, somewhat lumpy surface that lacks pinnae, instead the raised lumpy areas are slightly lighter in color but are smooth in texture. On the microscopical level fine hair-like protrusions cover the surface of the flap (Fig. 14D). These lateral flaps are held slightly away from the actual egg capsule, with the anterior margin simple and open, while the lateral flaps lateral margins are curled under slightly, but this rim does not rest on the egg capsule. A thin membrane runs along the inner side of the flap sealing the space between flap and capsule (Fig. 14H). The actual egg capsule lateral surface is slightly convex, with a smooth, only slightly lumpy surface (Fig. 14B). The dorsal surface has a thin micropylar plate which is ~½ the capsule length, but it is situated on the posterior 1/2 of the capsule (Fig. 12A). Running along each side of the micropylar plate is a continuous line of short, feather-like pinnae. The micropylar plate is thin, really only a slit with the widest portion around the small micropylar cup which is on the posterior ¼ of the capsule (Fig. 12A). These feather-like pinnae run fully around the sagittal plane of the egg, so when it is viewed laterally, the pinnae fully surround the capsule, with only the fused capitular frill projecting above the anterior pinnae (Fig. 12B). The operculum is ovular, and the outer margin has a fused capitular frill fully surrounding the opercular margin (Fig. 14C). This frill is fully fused around the entire margin and is prominently projecting above the flat, smooth surface of the cap, leaving only a narrow, long opening along the top of the egg (Fig. 12E). The fused capitular frill is roughly textured, appearing slightly fuzzy. The ventral surface of the egg capsule has the continuous encircling feather-like pinnae coming down from each side from the anterior, these two lines of pinnae gradually draw together until they fuse into one line near the posterior 1/3 of the capsule, where they continue on to the posterior (Fig. 12C). On the posterior, the encircling feather-like pinnae split around a central pinnae stalk with a feather-like apex (Fig. 12D).

##### Measurements including the extended pinnae [mm].

 Length (including operculum expansion): 7.6–7.7; maximum width of capsule when viewed from lateral aspect 4.8–4.9; length of micropylar plate 3.0–3.1.

##### Etymology.

Patronym; named to honor Frank Hennemann (Germany). At the time of this writing, Frank has named more than 350 phasmid species, reflecting decades of his dedicated work. Specifically within the phylliids, Frank’s 2009 publication (Hennemann et al. 2009) was instrumental in sparking the first author’s passion for leaf insects. At the time of its publication, Frank’s work was a major step towards revising the family and served as the foundation for many subsequent works on the group.

##### Distribution.

At present known from two provinces on Sulawesi, Indonesia (Fig. 2). The holotype locality of Bungadidi (South Sulawesi Province), and the paratype locality of Tiulapolu [= Tipulu] (Southeast Sulawesi Province).

##### Remarks.

The eggs of *Phyllium hennemanni* sp. nov. with their autapomorphic lateral flaps which do not react to humidity and are fixed at a small point on the bottom of the egg, maintain that the lateral flaps are held without touching the egg capsule itself (Fig. 12). Currently, the closest relative based on genetic sequences was recovered as *Phyllium mamasaense* (Fig. 1), a species which is sympatric and has adults with very similar morphology (such as femoral lobe shape and serration, genitalia, and coxae coloration). As with other phylliids, interestingly, despite adults being very morphologically similar, the eggs of these two closely related species differ drastically. At present, the adult male *Phyllium hennemanni* sp. nov. is still unknown, and once identified, may reveal further features for adult morphological differentiation.

#### ﻿ Phyllium illusorium

Taxon classificationAnimaliaPhasmatodeaPhylliidae

Cumming, Foley, Hennemann, Le Tirant & Büscher sp. nov.

AD89A2DC-707A-58F3-8F86-7AD3EC1594B5

https://zoobank.org/36E7C315-63F1-400D-929A-32A4CF378795

Fig. 15

##### Type material.

***Holotype*** (♂): Coll. I.R.SC.N.B.; Indonesia • Buton, xi.2012, Gift from B. Kneubuhler I.G.: 32.613. Tissue sample: SB0690 [RBINS]. ***Paratype*** (♂): INDONESIEN: S-Sulawesi Provinz, Sulawesi Tenggara, Buton Island, XI.2012. FH 0673-1 [Coll FH].

##### Differentiation.

Female, egg, and freshly hatched nymph unknown.

Male *Phyllium illusorium* sp. nov. are most similar to *Phyllium hausleithneri* and *Phyllium jacobsoni* due to similar femoral lobe shapes/serration, overall size and abdominal shape, and wing length/venation. *Phyllium illusorium* sp. nov. can be differentiated from *Phyllium hausleithneri* by the ventral coxae coloration, while subtle in males (and very prominent in females) *Phyllium hausleithneri* often have a slight purple hue to their ventral coxae surface, vs *Phyllium illusorium* sp. nov. which has white coxae. Male *Phyllium illusorium* sp. nov. are nearly indiscernible from male *Phyllium jacobsoni* as both species have white coxae and are very similar in most other regards (which is not surprising given their genetic close relation which was recovered in the phylogeny; Fig. 1). The only feature which allows consistent differentiation is the sagittal crest of the mesoprescutum which in *Phyllium illusorium* sp. nov. has prominent tubercles of a similar size to the anterior rim sagittal spine, vs *Phyllium jacobsoni* which only has small nodes along the sagittal crest. As with many phylliids, likely more prominent morphological differences are present in the egg or freshly hatched nymph stages, which are unfortunately unknown at the moment.

**Figure 15.**
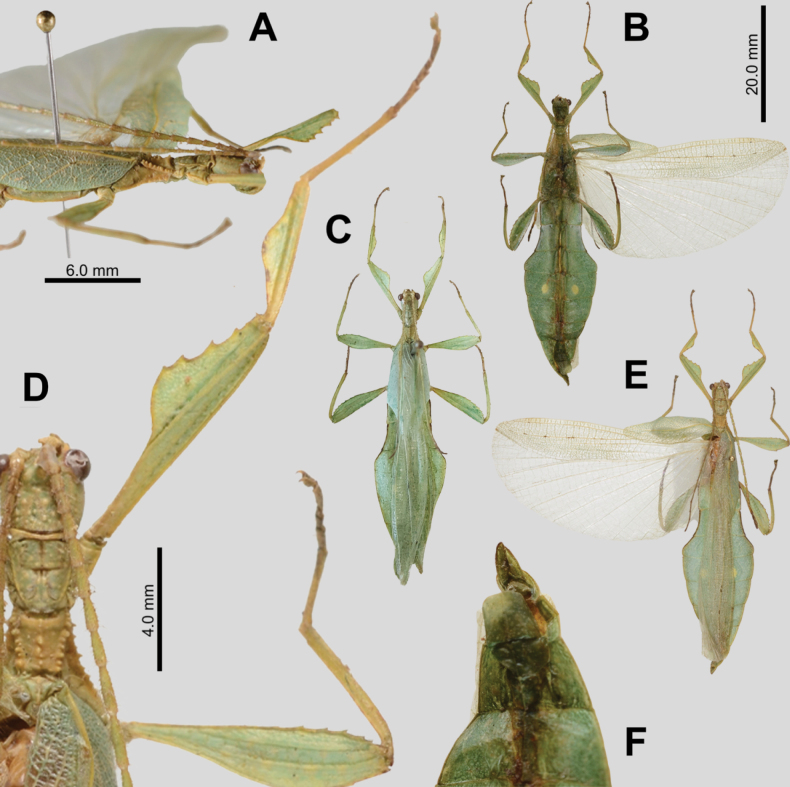
*Phyllium illusorium* sp. nov. holotype (B) [RBINS] and paratype (A, C, D, E, F) [Coll FH]. A. Detail of head and thorax, lateral; B. Holotype habitus, dorsal; C. Paratype habitus, ventral; D. Details of head, front leg, and thorax, dorsal; E. Terminalia, ventral; F. Paratype habitus, dorsal. Scale bar: 20.0 mm (B, C, E).

**Descripyion. Male. *Coloration*.** Coloration based upon the holotype and paratype specimens, which appear relatively well preserved (Fig. 15). Overall coloration pale green throughout with highlights of tan/reddish coloration on the margin of the protibial interior lobe, the lateral margins of abdominal segments II, III, and IV, and the tips of the antennae. Compound eyes are brown/reddish. The thorax and most of the antennae segments are straw yellow. On abdominal segment V are a pair of yellow eyespots. Ventral coxae coloration is white.

***Morphology*.***Head* capsule approximately as long as wide, with a vertex that is marked throughout with irregularly spaced and variably sized nodes. The posteromedial tubercle is singularly pointed but not particularly prominent (Fig. 15D). Frontal convexities are stout and bluntly pointed with sparse setae. Compound eyes large and bulbous, occupying ~2/5 of the head capsule lateral margins (Fig. 15D). There are three well-developed ocelli distinctly raised above the capsule and located between the compound eyes (Fig. 15D).

*Antennae* (including the scapus and pedicellus) consist of 23 segments, all segments except the scapus and pedicellus and terminal four segments are covered in dense setae where most are as long as or slightly longer than the antenna segment is wide. The terminal five segments are covered in dense short setae and the scapus and pedicellus are nearly completely bare with only a few sparse setae.

*Thorax*. Pronotum with anterior margin with a prominent rim which is slightly concave. Pronotum lateral margins are relatively straight and converge to a straight posterior margin that is ~½ the width of the anterior margin (Fig. 15D). Pronotum lateral margins have moderately formed rims and the posterior margin has a weakly formed rim (Fig. 15D). Face of the pronotum is marked by a distinct pit in the center, a sagittal furrow on the anterior ½, and slight perpendicular furrows originating from the central pit. The pronotum surface is marked throughout by low nodes (Fig. 15D). Prosternum and mesosternum surfaces are lumpy with distinctly formed nodes. Metasternum surface slightly wrinkled throughout, and marked with sparse granulation. Mesoprescutum approximately as long as wide, with lateral margins that are slightly converging to the posterior margin which is only slightly narrower than the anterior margin (Fig. 15D). Lateral margins of the mesoprescutum with eight or nine variably sized tubercles spaced unevenly, but all rather distinct (Fig. 15A). Mesoprescutum surface slightly raised along the sagittal plane which is marked with two distinct tubercles on the anterior 1/2 and two nodes on the posterior 1/2 (Fig. 15D). Mesoprescutum anterior rim distinctly raised and marked with a prominent sagittal spine; the remainder of the rim surface is relatively smooth (Fig. 15D). Mesopleurae begin on the anterior mesoprescutum margin and diverge at a gradually increasing angle from the anterior to the posterior but remain rather narrow throughout their length (Fig. 15D). Mesopleuron lateral margin with 6–8 moderately formed tubercles with one or two nodes between each set of tubercles (Fig. 15D). Mesopleuron face relatively smooth and marked by a distinct pit near the center and another near the anterior 1/3.

*Wings*. Tegmina moderate length, extending ½ of the way onto abdominal segment III. Tegmina wing venation: the subcosta (Sc) is the first vein, is simple, and terminates slightly less than 1/2 of the way through the overall wing length. The radius (R) spans the entire length of the tegmina with the first radius (R1) branching ~2/5 of the way through the wing length and terminates slightly more than halfway through the tegmina length, there is also a second radius (R2) which branches 1/3 of the way through the tegmina length and runs nearly directly to the tegmina margin. The radial sector, following these branchings, runs straight to the wing apex. The media (M) also spans the entire length of the tegmina running side by side along the radius/radial sector with only a vein or two width’s gap between them. The media posterior (MP) branches off near the middle of the tegmina and runs angled towards the apex/cubitus, and the media anterior (MA) runs straight to the tegmina apex. The cubitus (Cu) cuts across the tegmina to the margin ~1/3 of the way through the length and runs along the edge of the tegmina where the media posterior vein fuses with it and as the cubitus reaches the apex of the tegmina it fades. The first anal (1A) terminates upon reaching the cubitus ~1/3 of the way through the tegmina length. Alae well-developed in an oval fan configuration, long, reaching apical abdominal segment. Ala wing venation: the costa (C) is present along the entire foremargin giving stability to the wing. The subcosta (Sc) is short, fusing with the costa ~¼ of the way through the ala length. The radius (R) spans the entire wing and branches 2/5 of the way through the ala length into the first radius (R1) and radial sector (Rs) which run gently diverging for ~½ of their length, then run parallel until they near the apex where they converge slightly and terminate at the margin. The media (M) branches early, ~1/6 of the way through the ala length into the media anterior (MA) and the media posterior (MP) which run parallel with each other throughout the central 2/3 of the ala length, then the media posterior fuses with the media anterior and they run fused to join with the radial sector and this fused set of veins runs to the apex where it terminates. The cubitus (Cu) runs unbranched and terminates at the wing apex. Of the anterior anal veins, the first anterior anal (1AA) fuses with the cubitus near the ala base and then the first anterior anal branches from the cubitus 2/3 of the way through the ala length where it uniformly diverges from the cubitus until it terminates at the wing margin. The anterior anal veins 2–7 (2AA–7AA) have a common origin and run unbranched in a folding fan pattern to the wing margin. The posterior anal veins (1PA–6PA) share a common origin separate from the anterior anal veins and run unbranched to the wing margin with slightly thinner spacing than the anterior anal veins.

*Abdomen*. Lateral margins of abdominal segment II parallel; III diverging with increasing degree from the anterior to the posterior; segment IV diverging strongly for the anterior 2/3 to the widest point of the abdomen then running parallel for the posterior 1/3; V through X converging gradually with nearly smooth margins (at each suture the margins angle in very slightly). Overall, the abdomen has a spade-shaped appearance.

*Genitalia*. Poculum broad and ends in an apex that slightly passes the anterior margin of the abdominal segment X with a margin that is straight (Fig. 15F). Cerci long, slender, relatively uniform in width throughout their lengths, and with slightly more than ½ of their length extending from under abdominal segment X. The cerci are nearly flat and covered in a granulose surface with numerous short setae (Fig. 15F). Vomer broad and stout with straight sides evenly converging to the apical hook which is thick and has a singular point (Fig. 15F).

*Legs*. The profemoral exterior lobe is narrow, with a smooth margin, and slightly thinner than the profemoral shaft width. The profemoral interior lobe is obtusely triangular and at its greatest width it is ~1.5 × the greatest width of the profemoral shaft. The profemoral interior lobe is ornamented four serrate teeth of similar sizes and almost evenly spaced with looping gaps between them (Fig. 15D). Mesofemoral exterior lobe arcs end to end but is slightly wider on the distal 2/3 and on the distal ¼ it is marked with two small teeth, while the proximal remainder of the lobe lacks teeth. Mesofemoral interior lobe and the mesofemoral shaft are approx. the same width, while the mesofemoral exterior lobe is ~1.5 × wider than the mesofemoral shaft width. The mesofemoral interior lobe, is slightly broader on the distal end and the distal end is ornamented with six small, serrate teeth while the proximal portion of the lobe is thin and lacks teeth. Metafemoral exterior lobe has a straight margin running along the metafemoral shaft and is marked with only two small teeth near the distal ¼. Metafemoral interior lobe smoothly arcs end to end with nine sharply serrate teeth on the distal 2/3, which is wider than the smooth proximal portion of the lobe. Protibia lacking exterior lobe, interior lobe reaching end to end in a rounded triangle with the widest portion near the middle of the length; greatest width of the lobe is ~1.5 × the protibial shaft width; the proximal portion of the lobe is slightly thicker than the distal portion (Fig. 15D). Meso- and metatibiae simple, lacking lobes completely. The probasitarsus is slightly shorter than the protibial shaft length; the mesobasitarsus is slightly longer than ½ of the mesotarsus shaft length; and the metabasitarsus is slightly < ½ of the metatibial shaft length.

***Measurements of holotype male* [mm].** Length of body (including cerci and head, excluding antennae) 46.8, length of head 2.9, antennae 26.0, pronotum 2.6, mesonotum 2.2, length of tegmina 15.5, length of alae 37.4, profemora 9.0, mesofemora 8.5, metafemora 9.8, protibiae 6.1, mesotibiae 6.0, metatibiae 7.2.

***Measurements of paratype male* [mm].** Length of body (including cerci and head, excluding antennae) 49.0, antennae 27.6, pronotum 2.3, mesonotum 3.6, metanotum 3.9, length of tegmina 16.3, length of alae 37.0, greatest width of abdomen 12.3, profemora 9.2, mesofemora 8.8, metafemora 9.9, protibiae 5.8, mesotibiae 5.7, metatibiae 7.2.

##### Etymology.

The species epithet *illusorium* is the singular neuter form of the Latin *illusorius*, meaning mocking or ironical. While many leaf insects are colloquially described as “walking leaves,” *Phyllium illusorium* sp. nov. offers a subtle twist: it is not merely a leaf in motion, but rather a master of deception, a “mocking leaf” that plays with perception itself. Its intricate mimicry not only camouflages the insect among the foliage but also teases the observer, blurring the boundary between flora and fauna. The name celebrates this playful deceit, emphasizing the species’ role as a living illusionist within its arboreal habitat.

##### Distribution.

At present only known from the type locality; Buton Island, Southeast Sulawesi Province, Indonesia (Fig. 2).

##### Remarks.

At present this species is only known from the two type specimens from Buton island (Fig. 15). This species represents an interesting distribution as the other close members of its phylogenetically recovered clade (*Phyllium gardabagusi*, *Phyllium hausleithneri*, *Phyllium nisus*, and *Phyllium jacobsoni*) are all found west of Wallace’s line of faunal balance, while *Phyllium illusorium* sp. nov. is the first species from this clade found to the east (within Wallacea; Cumming et al. 2019a).

#### ﻿ Phyllium morganae

Taxon classificationAnimaliaPhasmatodeaPhylliidae

Cumming, Foley, Hennemann, Le Tirant & Büscher sp. nov.

3425B9B3-481E-5321-8DB0-6ECFBF22F0CC

https://zoobank.org/1A3DC9F8-B1A2-4529-89A2-6A9AC3DDC1C4

Fig. 16

##### Type material.

***Holotype*** (♀): Indonesia • Yapen isl., Kosiwo dist., Manainin vil., +/-1000m, 06/2022. Tissue sample SLT090 [IMQC]. Specimen received from Benny De Groof (Belgium), from his permitted export of Indonesian specimens.

##### Differentiation.

**Figure 16.**
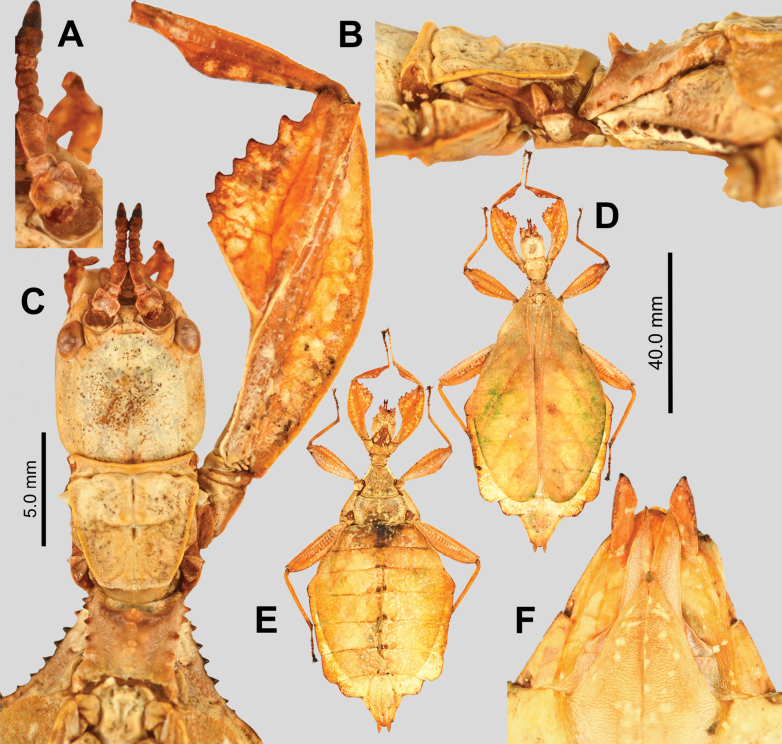
*Phyllium morganae* sp. nov. holotype female (IMQC). A. Detail of antenna, dorsal; B. Detail of head, antennae, front leg, and thorax, dorsal; C. Details of the thorax, lateral (head to the left); D. Habitus, dorsal; E. Habitus, ventral; F. Terminalia, ventral. Scale bar: 40.0 mm (D, E).

Male, egg, and freshly hatched nymph unknown. Female *Phyllium morganae* sp. nov. are most similar to *Phyllium philippinicum*Hennemann et al. 2009 and *Phyllium bilobatum* Gray, 1843 due to general abdominal shape with prominent lobes on segments VII and VIII. Many fine morphological details allow easy differentiation of these species. Likely the unknown female *Phyllium telnovi* has a similar morphology to *Phyllium morganae* sp. nov. as these were recovered as sister species (Fig. 1). Hopefully the female *Phyllium telnovi* can be located one day and allow proper differentiation. From *Phyllium philippinicum* the thorax morphology allows differentiation as *Phyllium philippinicum* has mesopleurae which are narrow on the anterior 1/3 vs *Phyllium morganae* sp. nov. has prominent mesopleurae which begin at the anterior margin of the mesothorax (Fig. 16C). Additionally, the uniform shape/sized teeth of the profemoral lobe interior in *Phyllium philippinicum* contrast with the large and variable sized/spaced teeth on the *Phyllium morganae* sp. nov. profemoral interior lobe (Fig. 16C). *Phyllium morganae* sp. nov. has profemoral interior lobes more similar to *Phyllium bilobatum* due to the variably sized/spaced large teeth, but the profemoral exterior lobe allows differentiation as the lobes are wider in *Phyllium bilobatum* (~2 × the width of the profemoral shaft), vs in *Phyllium morganae* sp. nov. which are thinner, only slightly wider than the profemoral shaft greatest width (Fig. 16C). The profemoral exterior lobe margin is also relatively smooth in *Phyllium morganae* sp. nov. but is distinctly granulate in *Phyllium bilobatum*.

##### Description.

**Female. *Coloration*.** Coloration description is based upon photographs of the live holotype specimen before it was preserved and dried (which resulted in most of the color being lost). The general coloration was lime green throughout, with the antennae brown, the larger veins of the tegmina dark orange/brown, and a few tan/brown patches present on the profemoral interior lobes and the mesofemoral lobes on the distal ends.

***Morphology*. *Head*** capsule slightly longer than wide, with a vertex that is slightly lumpy, not perfectly smooth (Fig. 16C). The posteromedial tubercle is present, singularly lobed, but not very prominent (Fig. 16C). Frontal convexity not very prominent and ending in a blunted point with several short setae across the surface. Compound eyes slightly protruding from the head capsule, not bulbous, taking up slightly < 1/3 of the head capsule lateral margins (Fig. 16C). Ocelli absent. Antennal fields slightly wider than the first antennomere width.

***Antennae*** consist of nine segments, with segment VIII slightly wider than segments VII or IX. The terminal antennomere is not particularly long, only slightly longer than the preceding segment. Antennomeres I–VII are smooth, and sparsely marked with short setae, the terminal two antennomeres are covered in short, dense setae, giving these segments a fuzzy appearance (Fig. 16A).

***Thorax*.** Pronotum with slightly concave anterior margin and lateral margins that converge only slightly, to the posterior margin which is slightly more than ½ the width of the anterior margin (Fig. 16A). The pronotum anterior margin has a prominent rim, while the lateral and posterior margins are less prominent. The pronotum surface is relatively smooth, with a prominent sagittal slit in the center and a few furrows lateral to this slit, and a prominent sagittal slit near the anterior margin (Fig. 16A). Prosternum and the anterior 1/3 of the mesosternum are marked by sparse, but prominent nodes, while the rest of the ventral thorax surfaces are relatively smooth. Mesoprescutum approximately as long as wide, lateral rims with five prominent tubercles (Fig. 16C). Mesoprescutum anterior rim prominently raised into a raised, broad sagittal spine (Fig. 16B). Mesoprescutum surface smooth except for the slightly raised mesoprescutum sagittal crest which is marked with at least two distinctly raised nodes; areas lateral to the sagittal crest smooth or slightly wrinkled (Fig. 16C). Mesopleurae begin on the anterior margin and diverge with nearly straight margins gradually (Fig. 16C). Mesopleurae lateral margins with five or six distinct tubercles which are relatively evenly spaced, and some of these have a node between them (Fig. 16C). Face of the mesopleura slightly wrinkled, with two notable divots, one on the anterior 1/3 and one near the posterior 1/3 (Fig. 16C).

***Wings*.** Tegmina long, reaching onto abdominal segment VIII. Tegmina venation; the subcosta (Sc) is the first vein in the forewing, running parallel with the margin for the first ½, and then bending and running towards the margin. The subcosta runs for ~1/5 of the tegmina length. The radius (R) spans the anterior ½ of the forewing with two subparallel branched veins; the first radius (R1) branches ~1/5 of the way through the wing length and terminates ~1/3 of the way through the tegmina length; the radial sector (Rs) branches ~1/3 of the way through the wing length and terminates near the distal 2/5 of the wing length. There is a continuation of the radius following the prominent Rs branching which continues on as a short but distinct R–M crossvein that connects the two veins. The media (M) is bifurcate with the media anterior (MA) terminating near the distal 1/5 of the tegmina and media posterior (MP) terminating near to the apex of the tegmina. There is a weak continuation of the media following the prominent media posterior (MP) branching which continues on as a somewhat long M–Cu crossvein that weakly connects the two veins. The cubitus (Cu) is also bifurcate, branching near the apex of the tegmina into the cubitus anterior (CuA) and cubitus posterior (CuP) which both terminate near the wing apex. The first anal vein (1A) is simple and fuses with the cubitus ~¼ of the way through the tegmina length. Alae vestigial.

***Abdomen*.** Abdominal segments II through the anterior 2/3 of IV gradually diverging. The posterior 1/3 of segment IV through the anterior 2/3 of segment VII are slightly and uniformly converging. The posterior 1/3 of segment VII is rounded inwards towards segment VIII which like VII starts converging gradually and then rounds inward to segment IX. Segments IX and X have straight, converging margins ending in a broad rounded apex (Fig. 16F).

***Genitalia*.** Subgenital plate starts at the anterior margin of tergum VIII, is broad and triangular, with straight margins, and extends ~1/2 of the way onto tergum X (Fig. 16F). Gonapophyses VIII are long and moderately broad, reaching the apex of the abdominal tergum X; gonapophyses IX are obstructed from view (Fig. 16F). Cerci flat, slightly broadening to the apical 1/3, with a slightly granular surface (Fig. 16F).

***Legs*.** Profemoral exterior lobe thin and arching from end to end gently, with a maximum width only ~1.5 × the greatest width of the profemoral shaft (Fig. 16C). Margin of the profemoral exterior lobe smooth or with slight granulation. Profemoral interior lobe ~2 × as wide as the greatest width of the profemoral shaft, and marked with six or seven variably sized teeth with looping gaps between them of varying widths (Fig. 16C). Mesofemoral interior lobe slightly thicker on the distal end, with the greatest width similar in wider to the mesofemoral shaft. The mesofemoral exterior lobes greatest width is also approx. as wide as the mesofemoral shaft width, but the weighting is towards the center, with the proximal and distal ends thin. The mesofemoral exterior lobe has one or two small, distally pointing teeth on the distal 1/3 of the lobe. The mesofemoral interior lobe has eight or nine small, distally pointing teeth on the distal ½ of the lobe. Metafemoral interior lobe arcs end to end, with the distal ½ slightly wider than the proximal ½ and marked with 12 or 13 small, serrate teeth on the distal ½ of the lobe. Metafemoral exterior lobe with only two or three very small teeth on the distal 1/3 and has a width similar to the metafemoral shaft width. Protibiae exterior simple, lacking a lobe. Protibiae interior lobe spans the entire length of the protibiae and is only slightly wider than the width of the protibiae shaft itself. The lobe is roundly triangular with the widest portion slightly situated on the distal ½. Mesotibiae and metatibiae simple, lacking exterior and interior lobes.

***Measurements*** (mm). Holotype, female: body length (including cerci and head, excluding antennae): 79.6, length/width of head: 7.7/6.0, antennae: 5.2, pronotum: 5.6, mesonotum: 6.5, length of tegmina: 51.0, greatest width of abdomen: 37.3, profemora: 15.6, mesofemora: 14.7, metafemora: 18.1, protibia: 9.6, mesotibia: 9.8, metatibia: 16.0.

##### Etymology.

Eponym; named to honor Morgan Brock-Smith (USA), recent wife to the first author. None of the hundreds of hours of research that has been focused on the phylliids over the years by the first author would have been possible without her support and love. Morgan is the stalwart partner that makes the adventures of life exciting and the challenges of life possible to overcome.

##### Distribution.

At present only known from the type locality of Yapen Island, Papua Province, Indonesia (Fig. 2).

#### ﻿ Phyllium ouelleti

Taxon classificationAnimaliaPhasmatodeaPhylliidae

Cumming, Foley, Hennemann, Le Tirant & Büscher sp. nov.

95185840-172C-59A6-AE6C-CDDDBBC7B753

https://zoobank.org/3BE98FC0-ECE2-4AE8-8BB2-564693EC4DBB

Fig. 17

##### Type material.

***Holotype*** (♀): Indonesia, North Maluku, Obi Island; VII-2021 [IMQC].

##### Differentiation.

**Figure 17.**
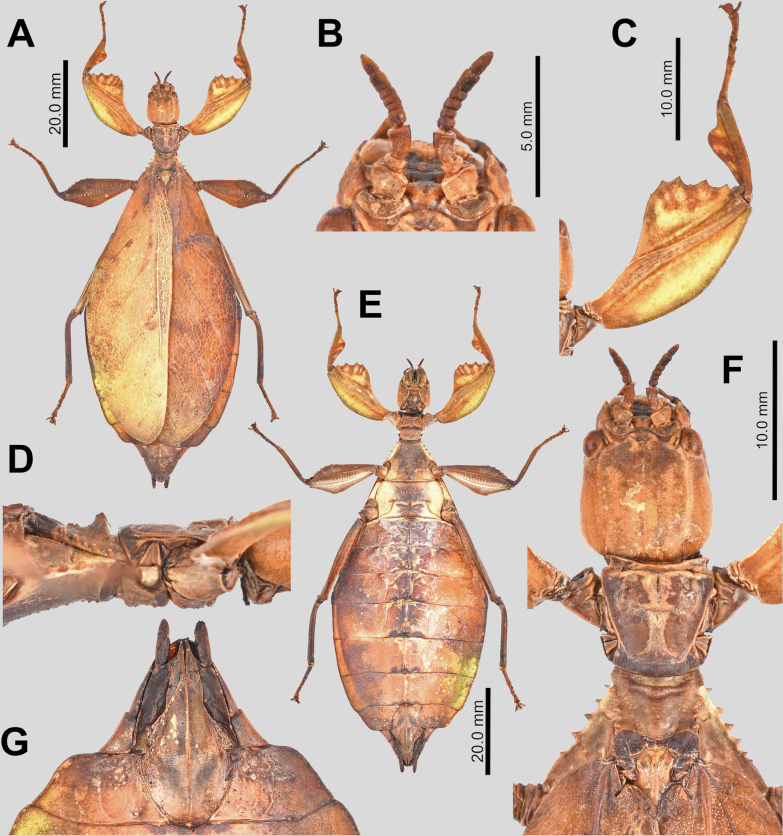
*Phyllium ouelleti* sp. nov. holotype female (IMQC). A. Habitus, dorsal; B. Details of antennae, dorsal; C. Detail of front right leg, dorsal; D. Thorax, lateral (head to the right); E. Habitus, ventral; F. Details of the antennae, head, and thorax, dorsal; G. Terminalia, ventral. Scale bars: 20.0 mm (A); 5.0 mm (B); 10 mm (C); 20.0 mm (E); 10.0 mm (F).

Presently only the female is known. The female *Phyllium ouelleti* sp. nov. is morphologically most similar to *Phyllium tobeloense* due to general femoral lobe shape/spination, coxae coloration, and overall body shape/size. The tegmina length and venation allows differentiation of these two species, as *Phyllium ouelleti* sp. nov. has longer tegmina, reaching the anterior margin of abdominal segment IX while *Phyllium tobeloense* has them only reaching onto segments VII or VIII. Likely due to the longer tegmina, there is also a difference in the venation as the media (M) is trifurcate in *Phyllium ouelleti* sp. nov. but bifurcate in *Phyllium tobeloense*. Also, these two species can be differentiated by the mesoprescutum surface area, as *Phyllium ouelleti* sp. nov. has a smaller surface due to a wider membranous attachment area of the forewings (vs *Phyllium tobeloense* which has smaller forewing attachment membranous areas, allowing the mesoprescutum to reach further back posteriorly). This membranous tegmina attachment area in *Phyllium ouelleti* sp. nov. also shortens the mesoprescutum lateral margins, resulting in the tubercles along these margins to be more tightly packed (Fig. 17F), vs in *Phyllium tobeloense* where they are slightly more spread out. Additionally, the mesopleurae lateral margin differs slightly, as *Phyllium ouelleti* sp. nov. has the margin nearly perfectly straight (Fig. 17F), vs *Phyllium tobeloense* which has them angled slightly more prominently inward towards the anterior, resulting in a slight bend on the anterior end. The tegmina length also differs as *Phyllium tobeloense* has shorter tegmina, only reaching to the anterior margin of abdominal tergite VIII or slightly onto it, but *Phyllium ouelleti* sp. nov. has tegmina which reach the anterior margin of tergite IX (Fig. 17A).

##### Description.

**Female. *Coloration*.** Coloration description is based upon the dead, dried holotype specimen (Fig. 17). Unfortunately, the holotype appears to have not been dried very well, and has many dark, discolored areas. Some coloration that can be seen is that the mesocoxae and metacoxae differ in color, with the mesocoxae a similar color to the surrounding tissue, while the metacoxae appear to have a dark spot, similar to *Phyllium tobeloense* but not quite as crisply edged as in that species. Additionally, the profemoral interior lobes and protibiae interior lobes appear to have some orange striping, a feature which is known from several *Phyllium* species, but is often variable in its intensity or completely absent in some individuals.

***Morphology*. *Head*** capsule is longer than wide, with a vertex that is relatively smooth except for a singularly pointed posteromedial tubercle which is not very large (Fig. 17F). Frontal convexity broad and ending in a relatively fine point; surfaces smooth except for a few short setae. Compound eyes only slightly protruding from the head capsule, not overly large, taking up ~¼ of the head capsule lateral margins (Fig. 17F). Ocelli absent. Antennal field slightly wider than the first antennomere.

***Antennae*** consist of nine segments, with the terminal segment approx. the same length as the preceding two segments’ lengths combined (Fig. 17B). Antennomeres I–VII smooth but sparsely marked with small transparent setae, the terminal two antennomeres are covered in dense, short setae giving them a fuzzy appearance (Fig. 17B).

***Thorax*.** Pronotum with slight concave anterior margin and straight lateral margins, which converge to the posterior margin that is ½ the width of the anterior margin (Fig. 17F). The pronotum surface is relatively smooth, with only a prominent pit in the center and a sagittal furrow on the anterior 1/2 (Fig. 17F). The pronotum has a distinctly formed anterior rim, moderately formed lateral rims, and a weakly formed posterior rim (Fig. 17F). Prosternum, mesosternum, and metanotum have nodes running along the sagittal plane, with the lateral margins relatively smooth, lacking nodes. Mesoprescutum approx. as long as wide, lateral rims with four tubercles and one or two smaller nodes interspersed (Fig. 17F). The tegmina membranous attachment areas are large and encroach on the mesoprescutum lateral margins, shortening them and compressing the tubercles somewhat (Fig. 17F). Mesoprescutum anterior rim prominent and raised into a blunted sagittal spine; the mesoprescutum rim surface is slightly lumpy (Fig. 17D). Mesoprescutum raised slightly along the sagittal crest, which is marked with three small nodes, while the remainder of the surface is smooth (Fig. 17F). Mesopleurae begin near the anterior rim, have straight margins, and run uniformly diverging throughout their lengths (Fig. 17F). Mesopleurae lateral margins with five or six moderately sized tubercles with minimal granulation between a few of the larger ones (Fig. 17F). Face of the mesopleura smooth or slightly wrinkled, with two notable divots, one on the anterior 1/3 and one less prominent one slightly posterior to the middle (Fig. 17F).

***Wings*.** Tegmina long, reaching the anterior margin of abdominal segment IX. Tegmina venation; the subcosta (Sc) is the first vein in the forewing, running parallel with the margin for the first 1/2, and then bending and running towards the margin where it terminates 1/3 of the way through the wing length. The radius (R) spans the central portion of the forewing with two subparallel branched veins; the first radius (R1) branches ~1/5 of the way through the wing length and terminates slightly proximal to the midline, and the radial sector (Rs) branches ~1/3 of the way through the wing length and terminates near the distal 1/3 of the wing length. There is a weak continuation of the radius following the prominent Rs branching which continues on as a short and thin R–M crossvein that does not appear to actually connect the two veins. The media (M) is trifurcate with a media anterior (MA; originating near the middle of the tegmina length and terminating on the distal 1/5), first media posterior (MP1; originating on the distal 1/3 of the tegmina length and terminating near the apex), and a second media posterior (MP2; which is small, originates near the distal 1/5 of the tegmina length and terminates near the tegmina apex). The cubitus (Cu) is also bifurcate, branching near the posterior 1⁄10 of the wing into the cubitus anterior (CuA) and cubitus posterior (CuP) which both terminate at the wing apex. The first anal vein (1A) is simple and fuses with the cubitus early on, ~¼ of the way through the tegmina length. Alae vestigial.

***Abdomen*.** Abdominal segments II through the anterior 2/3 of IV uniformly diverging. The posterior 1/3 of segment IV through segment VII are converging, with the degree of convergence slightly increasing from IV through VII. The posterior 1/3 of segment VIII ends in a slightly rounded lobe. Segments IX and X are notably narrower than the previous segments, and have straight, converging margins to the broad rounded apex.

***Genitalia*.** Subgenital plate starts at the anterior margin of tergum VIII, is moderately broad, and extends halfway onto tergum X with straight margins ending in a fine point (Fig. 17G). Gonapophyses VIII are long and moderately broad, reaching the apex of abdominal tergum X; gonapophyses IX are shorter and narrower, hidden below gonapophyses VIII (Fig. 17G). Cerci flat, somewhat rectangular, with a slightly rough textured surface (Fig. 17G).

***Legs*.** Profemoral exterior lobe smoothly arching from end to end, ~3 × wider than the width of the profemoral shaft greatest width (Fig. 17C). Edge of the profemoral exterior lobe smooth and unadorned (Fig. 17C). Profemoral interior lobe as wide as the exterior lobe, approximately right angled, and marked with six teeth (four large and two small) arranged in a small-large-large-small-large-large pattern, with looping gaps between the teeth (Fig. 17C). Mesofemoral exterior lobe roughly a narrow, rounded triangle with the greatest width only slightly wider than the mesofemoral shaft width, and the greatest width situated on the distal 1/3 of the mesofemora. Just distal to the mesofemoral exterior lobe greatest width is a singular, small tooth. Mesofemoral interior lobe is approx. the same width as the mesofemoral shaft, and is similar to the exterior lobe with the greatest width on the distal 1/3, but the interior lobe is slightly more rounded, not as straight edged as the exterior lobe. Mesofemoral interior lobe distal 1/2 is also marked by six or seven small, distally pointing, serrate teeth. Metafemoral interior lobe arcs end to end, with the distal 1/2 slightly wider than the proximal 1/2 and marked with nine serrated teeth on the distal 1/2 of the lobe. Metafemoral exterior lobe is thin and smooth, hugging the metafemoral shaft and lacks dentation. Protibia lacking an exterior lobe (Fig. 17C). Protibia interior lobe spans the entire length of the protibia and is ~2 × the width of the protibia shaft itself. The lobe is roundly triangular with the widest portion on the distal 1/2. Mesotibiae and metatibiae simple, lacking exterior and interior lobes.

***Measurements*** (mm). Holotype, female: body length (including cerci and head, excluding antennae): 93.5, length/width of head: 10.5/8.1, antennae: 5.3, pronotum: 6.7, mesonotum: 7.8, length of tegmina: 62.6, greatest width of abdomen: 37.0, profemora: 18.8, mesofemora: 16.5, metafemora: 22.7, protibia: 11.3, mesotibia: 11.7, metatibia: 17.8.

##### Etymology.

Patronym, named after Pierre-Olivier Ouellet, who donated the specimen to the IMQC.

##### Distribution.

At present only known from Obi Island, Indonesia (Fig. 2).

##### Remarks.

Despite Obi being a relatively small island (ca 2,542 km^2^), this new species represents the second species of leaf insect known from Obi Island, the first being *Comptaphyllium regina* (Cumming et al. 2019b). Morphologically this species is most similar to *Phyllium tobeloense* and likely represents a closely related species. Unfortunately, the holotype *Phyllium ouelleti* sp. nov. is somewhat degraded and was therefore not included in our molecular phylogeny to confirm this relationship.

## ﻿Discussion

The following discussion integrates morphological, ecological, and biogeographic insights arising from our analysis of several newly described *Phyllium* species within the greater context of the *Phyllium* of Indonesia. We highlight novel and previously undocumented egg morphologies, discuss their potential adaptive significance, and draw comparisons with known structures in other taxa. In addition, we examine patterns of species distribution and diversification across Southeast Asia and the Indo-Australian Archipelago, interpreting them through the lens of historical land connections and biogeographic boundaries. Together, these findings contribute to a deeper understanding of phylliid evolution and the complex biogeographic history of this highly fragmented region.

Egg morphology in Phylliidae is remarkably diverse and often highly species-specific, providing valuable characters for taxonomic identification and differentiation (Büscher et al. 2023). While adult morphology is relatively conserved across taxa, egg traits—such as size, shape, chorion structure, pinnae arrangement, and adhesive adaptations—vary significantly across species (Cumming et al. 2019a, 2020a). These features have been especially informative in past studies, playing a central role in resolving species boundaries and contributing to a more refined understanding of phylliid diversity.

*Phyllium hennemanni* sp. nov. eggs (Figs 12, 14) present a wholly unique morphology both within phylliids and for the greater Phasmatodea. Upon realizing that the lateral surfaces of the egg were in fact flaps, not just the solid, bald surface of the capsule, the eggs were exposed to various moisture levels to determine if the flaps would move/react to contact with water (as can happen with pinnae on other *Phyllium* eggs; Büscher et al. 2023). Despite various humidities and being dried/rehydrated multiple times, the flaps appeared fixed in place, held slightly away from the egg capsule surface. This position maintains a slight pocket of air around the egg capsule, the purpose of which can presently only be speculated at. One potential function of these lateral flaps may be protection against parasitoids, given that parasitoid wasps are hypothesized to be a key driver for the evolution of phasmatodean egg morphology (Goldberg et al. 2015; Robertson et al. 2018). These lateral flaps on the egg represent a previously unknown egg morphotype within the phylliids (Büscher et al. 2023). While all *Phyllium* have some form of adhesion and pinnation present (with most *Phyllium* having “type 5” pinnae as described in Büscher et al. (2023) as being “feather-like and hierarchically splitting pinnae with a broad base and several side branches”), a few species of *Phyllium* (*Phyllium bonifacioi*, *Phyllium ericoriai*, and *Phyllium mamasaense*) have “type 4” pinnae (defined by Büscher et al. (2023) as “short and dispersed clusters of primitive pinnae on solid ridges”). However, the reinforced ribs in *Phyllium ericoriai* and *Phyllium bonifacioi* differ from those in *Phyllium mamasaense* due to their origin. While the ribs of *Phyllium ericoriai* and *Phyllium bonifacioi* are structural elevations protruding from the remaining egg surface (Büscher et al. 2023), those of *Phyllium mamasaense* arise from deep furrows between longitudinal rows of pinnae. The flaps of *Phyllium hennemanni* sp. nov. are likely formed by the same kind of invagination of the chorion as in *Phyllium mamasaense* which fused and formed a shield-like flap surrounding the remaining capsule. Similar chorion expansions are known from *Trachythorax* spp. (Bresseel and Constant 2021) where it has been interpreted as a counteradaptation against parasitoids. In *Trachythorax albomaculatus* Bresseel & Constant, 2021 the chorionic structures expand after oviposition and react to the humidity of the environment and may shield the vulnerable embryo within the egg from the parasitoid by establishing a barrier. This spacing might prevent the parasitoid from reaching the embryo for oviposition. The egg expansions in *Trachythorax albomaculatus* are additionally covered by a fine crystalline powder (Büscher et al. 2024) that might additionally contaminate the adhesive systems of the parasitoids to prevent them from gaining foothold on the egg. The large flaps of *Phyllium hennemanni* sp. nov. can likewise prevent parasitoids from ovipositing into the egg, as the spacing beneath the flap increases the distance to the embryo and increases the effort of the parasitoid to lay its egg into the actual capsule.

The eggs of *Phyllium cayabyabi* sp. nov. present yet another previously unknown egg morphotype (Büscher et al. 2023). Their autapomorphic spatulate pinnae carry glue (Fig. 8) which likely functions similar to the adhesive mechanisms found in other *Phyllium* spp. to attach the egg in beneficial environmental conditions for incubation (Büscher et al. 2020a, 2020b). Different pinnae morphologies in Phylliidae are adapted to various surface conditions (Büscher et al. 2023). The specialized morphology of the pinnae of *Phyllium cayabyabi* sp. nov. increases the surface for adhesion, but the dense coverage of the two pinnae types on this egg furthermore enables mechanical interlocking with structured substrates by the collective effect of a number of pinnae. Furthermore, the minor chorionic outgrowths on the pinnae extend the functionality of the adhesive system of this species with mechanical interlocking. The outgrowths likely provide enhanced grip on rough substrates, even if no glue is activated.

Biogeographically, Indonesia is a deeply interesting region for *Phyllium* as it is likely the origin of the clade (Bank et al. 2021b) and the Pleistocene island aggregates that resulted from the rise and fall of sea levels have influenced the distribution patterns and subsequent speciation in this region. Our results show at least five independent colonization events of the Indonesian archipelago (see blue geographic labels within the tree of Fig. 1): three to Kalimantan, potentially from the Philippines and Sabah (Borneo, Malaysia), one to Wallacea (*Phyllium siccifolium*-group) from the Philippines, and another to Wallacea (Halmahera) with subsequent dispersal to Australasian Papua and Yapen as well as Java, Sumatra, and Buton.

Within Wallacea, we find one clade including *Phyllium tobeloense*, *Phyllium bankae*, and likely *Phyllium ouelleti* sp. nov. (not included in molecular analysis but morphologically closest to *Phyllium tobeloense*), which is restricted to islands between Weber’s and Lydekker’s lines—Morotai, Halmahera, and Obi. A second clade comprises *Phyllium siccifolium*, *Phyllium letiranti*, *Phyllium mamasaense*, and *Phyllium hennemanni* sp. nov. While *Phyllium siccifolium* occurs east of Weber’s line (on the relatively small and closely situated islands of Buru, Ambon, and Seram), the other three species are found to the west on Sulawesi and its satellite islands. The geographic complexity of Sulawesi has likely promoted repeated speciation within this clade (Lohman et al. 2011; Mcguire et al. 2023).

In contrast to these geographically rather restricted Wallacean clades, other lineages span wider regions as, for instance, the one including *Phyllium gardabagusi*, *Phyllium hausleithneri*, *Phyllium nisus*, and *Phyllium jacobsoni*—species exclusively found west of Wallace’s Line. This clade, however, also contains *Phyllium illusorium* sp. nov., which is the only relative known to occur east of the line, indicating a rare eastward dispersal across the line of faunal balance.

*Phyllium morganae* sp. nov., from Yapen Island, was recovered as sister to *Phyllium telnovi*, found on mainland West Papua, which possibly represents an island speciation event. These are among the few *Phyllium* species recorded east of Lydekker’s Line (*Phyllium elegans* being the third). However, the phylogenetic placement of *Phyllium telnovi* and *Phyllium morganae* remains uncertain. Bank et al. (2021b) recovered *Phyllium telnovi* as being related to a southern Philippines clade (UFBoot = 88), while this study recovers *Phyllium telnovi* and *Phyllium morganae* as related to a West Indonesian/Malay Peninsula clade (support value SH-aLRT = 92.8, UFBoot = 65; Fig. 1). These Australasian species may reflect long-distance dispersal from early-diverging lineages, though further sampling across New Guinea is needed to clarify their phylogenetic position.

*Phyllium crapulatum* sp. nov. belongs to a clade composed of an unidentified nymph from Mount Bawang (“*Phyllium* sp. 5” from Bank et al. 2021b) and *Phyllium rubrum* from Peninsular Malaysia. This distribution aligns with the historical Sunda Shelf land connections (Sundaland), which facilitated biotic exchange during Pleistocene low sea levels (ca 2.6 mya; Voris 2000; Lohman et al. 2011). While some phylliid taxa such as *Pulchriphyllium bioculatum* and *Pulchriphyllium shurei*, show broad yet genetically cohesive distributions across this region, the *Phyllium crapulatum*–*Phyllium rubrum* clade exhibits clear genetic and morphological divergence.

Lastly, *Phyllium cayabyabi* sp. nov. and *Phyllium boislardi* sp. nov. belong to two separate clades that show a comparable biogeographic pattern: each contains one species isolated on Palawan and a second, more diverse branch on Borneo. These patterns likely reflect historical connectivity via the Pleistocene land bridge (ca 2.6 million–11,700 years ago) between Borneo and Palawan (Cheng and Faidi 2025), enabling dispersal followed by allopatric divergence.

The observed patterns of distribution and diversification within *Phyllium* underscore the evolutionary influence of Southeast Asia’s dynamic geological and ecological history. The presence of multiple independent colonization events into and within Wallacea, the discovery of island-endemic lineages, and evidence of both recent and ancient divergence events highlight how even subtle geographic barriers have played a critical role in shaping species-level diversity. These findings not only contribute to our understanding of phylliid biogeography but also emphasize the importance of integrating molecular, morphological, and geographical data in taxonomic studies of cryptic organisms. Moreover, given ongoing habitat fragmentation and deforestation in many parts of the Indo-Australian Archipelago, these results stress the urgency of documenting and conserving narrowly distributed taxa before further biodiversity loss occurs. Continued sampling across underexplored islands and regions will be essential for resolving remaining phylogenetic uncertainties and uncovering the full extent of hidden diversity within *Phyllium*.

## Supplementary Material

XML Treatment for Phyllium

XML Treatment for Phyllium boislardi

XML Treatment for Phyllium cayabyabi

XML Treatment for Phyllium crapulatum

XML Treatment for Phyllium hennemanni

XML Treatment for Phyllium illusorium

XML Treatment for Phyllium morganae

XML Treatment for Phyllium ouelleti

## ﻿Additional information

### Use of AI

No use of AI was reported.

### Author ORCIDs

Royce T. Cumming https://orcid.org/0000-0001-7930-1292

Evelyn Marie Foley https://orcid.org/0009-0007-9754-212X

Daawia https://orcid.org/0000-0003-4851-0862

Bambang Suhartawan https://orcid.org/0009-0005-2162-0246

Katharina Henze https://orcid.org/0009-0007-5474-1276

Thies H. Büscher https://orcid.org/0000-0003-0639-4699

Sarah Bank https://orcid.org/0000-0001-6952-1590

### Data availability

All of the data that support the findings of this study are available in the main text or Supplementary Information.

## Supplementary materials

Supplementary material 1 All sampled specimens are listed with their collection data, deposition location, and GenBank accession numbers for the generated sequences Data type xlsx Explanation note Taxon information for each specimen used in the phylogenetic analysis including sex, type designation, sample ID, sampling site, and GenBank accession numbers. Newly generated molecular data in bold. List of species included for the phylogenetic analysis. Information and sampling were taken from Bank et al. (2021b). Taxonomic changes are highlighted by asterisks and newly added specimens are in bold. Material from type species indicated as HT = holotype, NT = neotype and PT = paratype. Accession numbers are listed for each sequence used (16S, 18S, 28S, COI, COII, H3), while newly sequenced data are in bold. Empty cells imply absence of available DNA sequence. Species in alphabetical order. https://binary.pensoft.net/file/1447236This dataset is made available under the Open Database License (http://opendatacommons.org/licenses/odbl/1.0/). The Open Database License (ODbL) is a license agreement intended to allow users to freely share, modify, and use this Dataset while maintaining this same freedom for others, provided that the original source and author(s) are credited.Royce T. Cumming, Sarah Bank

Supplementary material 2 Multiple sequence alignment of concatenated dataset Data type fas Explanation note Multiple sequence alignment including partitioning scheme in Nexus format for the concatenated dataset of the 58 taxa used for the phylogenetic analysis. https://binary.pensoft.net/file/1447237This dataset is made available under the Open Database License (http://opendatacommons.org/licenses/odbl/1.0/). The Open Database License (ODbL) is a license agreement intended to allow users to freely share, modify, and use this Dataset while maintaining this same freedom for others, provided that the original source and author(s) are credited.Sarah Bank

Supplementary material 3 Phylogenetic tree file in newick format Data type tre Explanation note Phylogenetic tree file in newick format. https://binary.pensoft.net/file/1447238This dataset is made available under the Open Database License (http://opendatacommons.org/licenses/odbl/1.0/). The Open Database License (ODbL) is a license agreement intended to allow users to freely share, modify, and use this Dataset while maintaining this same freedom for others, provided that the original source and author(s) are credited.Sarah Bank
